# Determining the parent and associated fragment formulae in mass spectrometry via the parent subformula graph

**DOI:** 10.1186/s13321-023-00776-y

**Published:** 2023-11-07

**Authors:** Sean Li, Björn Bohman, Gavin R. Flematti, Dylan Jayatilaka

**Affiliations:** 1 School of Molecular Sciences, The University of Western Australia, 35 Stirling Highway, Crawley, 6009 Australia; 2 Department of Plant Protection Biology, Swedish University of Agricultural Sciences, Box 190, 23422 Lomma, Sweden

**Keywords:** Mass spectrometry, Fragmentation, Molecular formula, Graph theory, Combinatorics, Natural products, Metabolomics

## Abstract

**Background:**

Identifying the molecular formula and fragmentation reactions of an unknown compound from its mass spectrum is crucial in areas such as natural product chemistry and metabolomics. We propose a method for identifying the correct candidate formula of an unidentified natural product from its mass spectrum. The method involves scoring the plausibility of parent candidate formulae based on a parent subformula graph (PSG), and two possible metrics relating to the number of edges in the PSG. This method is applicable to both electron-impact mass spectrometry (EI-MS) and tandem mass spectrometry (MS/MS) data. Additionally, this work introduces the two-dimensional fragmentation plot (2DFP) for visualizing PSGs.

**Results:**

Our results suggest that incorporating information regarding the edges of the PSG results in enhanced performance in correctly identifying parent formulae, in comparison to the more well-accepted “MS/MS score”, on the 2016 Computational Assessment of Small Molecule Identification (CASMI 2016) data set (76.3 vs 58.9% correct formula identification) and the Research Centre for Toxic Compounds in the Environment (RECETOX) data set (66.2% vs 59.4% correct formula identification). In the extension of our method to identify the correct candidate formula from complex EI-MS data of semiochemicals, our method again performed better (correct formula appearing in the top 4 candidates in 20/23 vs 7/23 cases) than the MS/MS score, and enables the rapid identification of both the correct parent ion mass and the correct parent formula with minimal expert intervention.

**Conclusion:**

Our method reliably identifies the correct parent formula even when the mass information is ambiguous. Furthermore, should parent formula identification be successful, the majority of associated fragment formulae can also be correctly identified. Our method can also identify the parent ion and its associated fragments in EI-MS spectra where the identity of the parent ion is unclear due to low quantities and overlapping compounds. Finally, our method does not inherently require empirical fitting of parameters or statistical learning, meaning it is easy to implement and extend upon.

**Scientific contribution:**

Developed, implemented and tested new metrics for assessing plausibility of candidate molecular formulae obtained from HR-MS data.

**Supplementary Information:**

The online version contains supplementary material available at 10.1186/s13321-023-00776-y.

## Introduction

Notwithstanding improvements in experimental instrumentation and technique, the determination of the molecular formula of an unknown compound from mass spectrometry (MS) data remains a key bottleneck to further analysis, especially if the molecular ion is in low abundance or overlapping with other unrelated fragments. To address this, we consider here a slightly more general problem: to annotate with molecular formula every mass peak (if possible) in the mass spectrum of low-to-medium mass compounds (Da $\le 1000$) —a process we call *whole-spectrum formula annotation*.

**Fig. 1:**
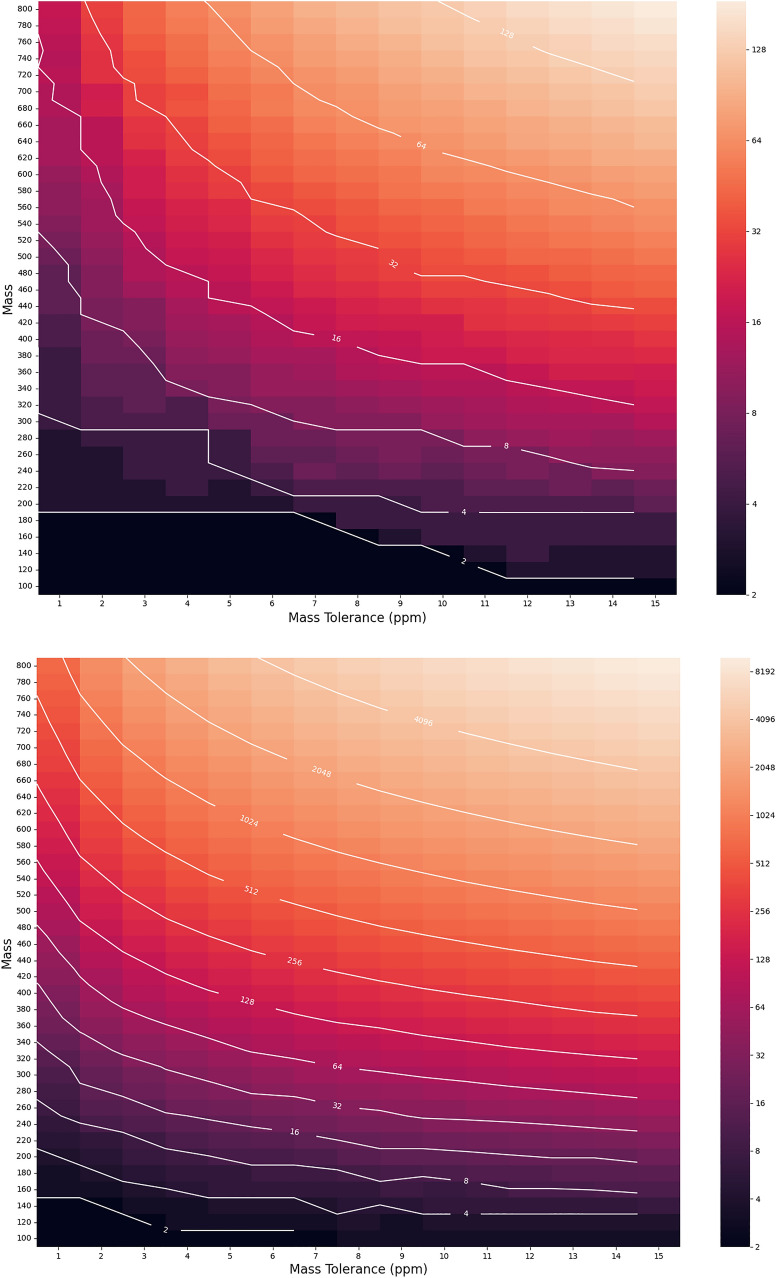
The number of molecular formulae as a function of mass tolerance of a mass spectrometer, assuming the element is made from an alphabet of elements C, H, N, O (above) and C, H, N, O, P, S (below), subject to the condition that the number of hydrogens in the formula is less or equal to twice the maximum number of carbon atoms assignable to the formula, and that each formula is at least 25% carbon by mass

Usually the unknown compound is present in a mixture; we assume so here. Then, the nature of the mass spectra obtained is tightly coupled to physico-chemical properties of that unknown—since these dictate both the kind of *separation techniques* used to purify it at the inlet of the mass spectrometer, (e.g. liquid chromatography (LC) or gas chromatography (GC)), and the *ionization method* used to form the parent ion in the spectrometer, (e.g. “hard” electron-impact (EI) ionization, or “soft” low-energy methods such as electrospray ionization (ESI), chemical ionization (CI), or electromagnetic field ionization [1]). Soft ionization methods are more likely to lead to the parent ion mass peak being observed in the spectrum, however such a spectrum generally does not contain many fragments, and consequently possesses less structural information. Therefore, putative parent ions from a soft first ionization stage are often fed into a second hard ionization stage, that yield more fragments [2, 3]. This is usually referred to as MS/MS or MS$^2$. Regardless of the separation method or ionization technique, if separation fails, or if the spectrum is noisy, the undesirable spectral components may be be removed via signal processing techniques [4–6]. Assuming these difficulties are overcome, there remains the problem of achieving sufficient resolution in the mass spectrometer to obtain the molecular formula of the analyte. Since the precision of mass spectral measurements are given in relative units, it follows that there will always be some analyte of sufficiently large mass, or composed of so many elements it becomes impossible to unambiguously determine its molecular formula from a given spectrometer. Figure 1 shows when the molecular formula may be obtained unambiguously as a function of resolution.

### Formula annotation via isotopic profile matching

If the error inherent to the mass measurement is too great to unambiguously resolve the molecular formula of the unknown parent molecule, then a general strategy is to leverage extra information in order to annotate the spectrum. The first additional source of information which can be utilised to disambiguate between the possibilities is the abundance of the the $M+1$ and $M+2$ isotopologue mass peaks [7], or even the $M+3$ mass peak [8], if these exist, relative to the monoisotopic mass peak. Grange and coworkers call this approach ion composition elucidation (ICE) via relative isotopic analysis (RIA) [7]. More generally, this idea is called  *isotopic profile matching*, which implies more complex isotopic envelope calculations and comparisons. These techniques are widely used for the elucidation of molecular formulae with high-resolution mass spectrometers [9–16].

### Formula annotation via additional experiments

In some cases, if it is viable to do so, experimental interventions alone may allow for the unambiguous determination of the parent molecular formula [17–20]. For example, one may use Fourier transform (FT) ion-cyclotron resonance (ICR), which allows for ultra-high resolution mass measurements [21] in order to determine the analyte molecular formula unambiguously. FT-ICR instruments allow for extremely accurate mass measurements; they can possess a resolution of one million or greater. However, FT-ICR instruments are extremely expensive and consequently out of reach of most laboratories. In contrast, due to the increasing availability and ease of use of benchtop tandem mass spectrometers such as Q-TOFs and Orbitraps, mass spectrometers that can record accurate mass measurements are becoming more accessible. In particular, in a general MS$^n$ experiment, which is possible with a tribrid Orbitrap mass spectrometer, the (fragment) ions comprising a mass peak may be fragmented again, offering the potential to systematically reconstruct molecular formula of the parent from that of its parts [22–26]. Unfortunately, such experiments generate large amounts of data, which complicates the analysis; and if there is insufficient amount of sample, the signal from successive fragmentation may be weak.

### Simple rule-based methods for formula annotation

Another source of such extra information comprises “rules of thumb”, derived empirically, not always correct, but can be used nonetheless to filter out unlikely candidate formulae. Such rules include constraints on the number and type of elements in a given molecular formula such that they can be drawn as sensible Lewis structures. Well known examples are the “nitrogen rule”, [27, 28], Senior’s three rules [29], and the rule that the ring and double-bond equivalent (RDBE) value of a molecular formula take on integer values of zero or more [30]. The Kendrick Mass Defect relative to integer-normalized-mass $\hbox {CH}_{2}$ groups [31], or other chemical groups or entities [32], may also be plotted, as in van Krevelen diagrams [33], which may also help molecular formula determination for specific classes of molecules [17]. By considering 400,000 compounds, Kind and Fiehn [8] derived “seven golden rules” that involve constraints of element valencies and elemental ratios. However, even here still 2% of known compounds do not satisfy all the rules; and the accuracy was even lower for “truly novel compounds”, which may be of greatest interest.

**Fig. 2:**
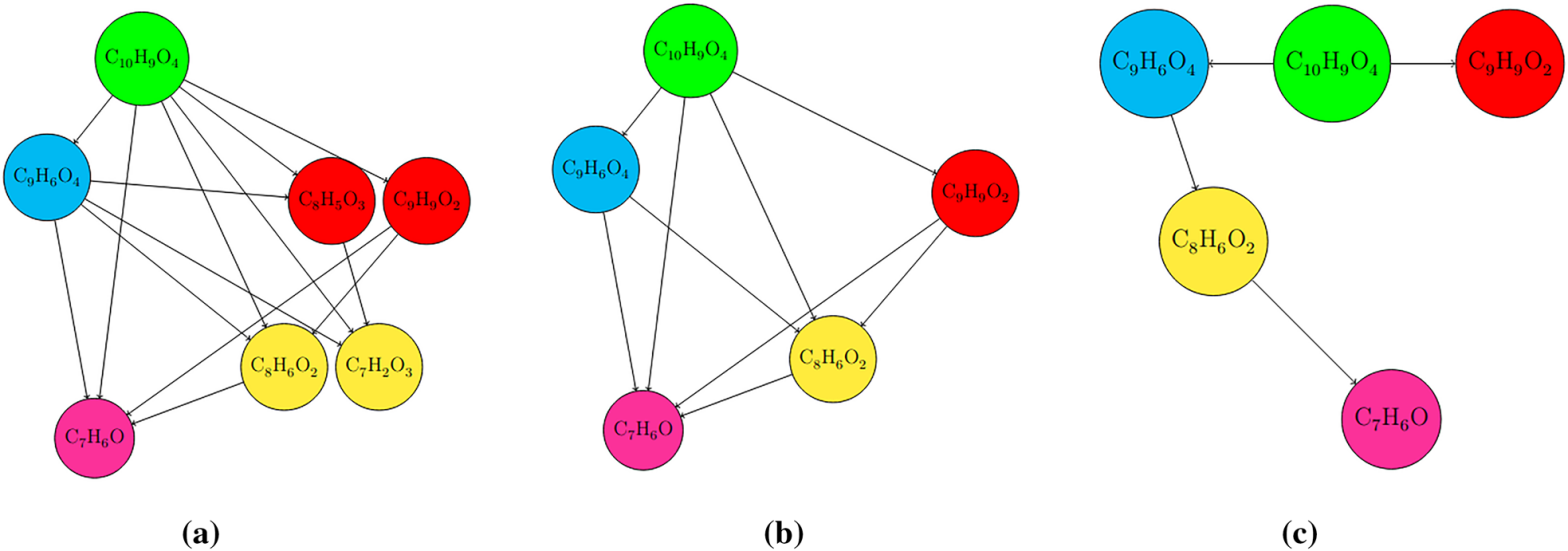
**a** Böcker and coworkers' fragmentation graph. Each vertex corresponds to a candidate formula for some mass in the mass spectrum, and vertices of the same color are candidate formulae of the same mass. Observe that there is no line between the $\hbox {C}_{7}\hbox {H}_{6}\hbox {O}$ and $\hbox {C}_{8}\hbox {H}_{5}\hbox {O}_{3}$ vertices, as they are not subformulae of each other. **b** A possible subformula graph, which is a subgraph of **a** and also explains the same mass spectrum encoded by (**a**). Note that all vertices possess distinct colours, which means masses in the spectrum are only assigned a maximum of one candidate formula. **c** A possible fragmentation tree (colourful subtree) of Böcker and coworkers. The vertices are also distinct colours, but each vertex only has one incoming edge

### Graph theoretical methods for formula annotation

In mass spectrometry, hard ionization primarily results in *unimolecular fragmentations*, where a compound breaks down into a cationic fragment and a neutral radical or stable molecule. These fragmentations can occur sequentially, leading to a reaction network that can be encoded as a directed acyclic graph (DAG). The graph’s vertices represent chemical species, and a directed edge encodes the relationship between parent and child fragments. Such reaction networks have been called “family trees” [34] and “identification trees” [35] in a mass spectral context, and the construction of such trees from mass spectra based on chemical knowledge enables the analyst to identify unknown analytes.

In general, one cannot conclude from (non-MS$^n$) mass spectra alone how fragment masses are related to each other at the level of the reaction network (e.g if one is a parent of the other, or if they are siblings that share a common parent ion). However, provided only fragmentation reactions occur, the formula of a child species must be a subformula of the formula of the parent species from which it is derived. This restriction allows one to construct a number of reaction network-like graphs, which also assist in the formula and/or structural elucidation process, even if they do not correspond to the true reaction network [24, 36, 37].

Although not necessarily graph-theoretical, an early example of the use of this restriction is by Grange and coworkers [7], who claimed that “determination of the unique composition of a fragment ion or a neutral loss requires less stringent error limits than for the [parent] ion” (see also [38]). From this, they proposed an “ion correlation” algorithm for $\mathrm {MS/MS}$ spectra, where for a given candidate formula of the precursor (parent) ion, candidate formulae for the most abundant fragment ions and corresponding neutral loss, were computed such that either the formula for the fragment or neutral loss species has masses within an acceptable tolerance *and* are subformula of the formula for the candidate parent species. If no corresponding formulae satisfying these conditions exist for the select fragment mass peaks of high intensity, then the parent candidate formula was ruled out. In this way, one can reduce the number of plausible candidate formulae for the parent ion.

Böcker and Rasche [36] subsequently proposed a related graph-theoretical method, representing the mass spectrum by “fragmentation trees” (FT), illustrated in Fig. 2c. The FT comprises vertices that represent the mass peaks in the spectrum, and are labelled by their putative molecular formulae, and edges are connected if the subformula relationship holds. Each FT is associated with a score, which depends on a particular whole-spectrum formula annotation. The FT with the highest score defines the best annotation. The score of the FT is calculated as the sum of vertex scores, with each vertex score being the logarithm of a product of terms involving the number of weak-intensity peaks, predicted peak-mass deviation, heteroatom-carbon ratios, energy-dependence of fragment and parent peak appearances, uncommon neutral losses, and large-mass neutral losses. This method was first developed for MS/MS spectra, but later was generalised to MS$^n$ [25] and EI-MS spectra [39]. The SIRIUS software package utilises this approach, combined with isotopic profile matching, to perform whole-spectrum annotation [40].

Pluskal and coworkers [14] proposed a very similar idea to that of Grange and coworkers, also for MS/MS spectra. Rather than picking only the most highly abundant mass peaks, they instead filtered out parent candidate formulae by calculating the score, which they refer to as the “MS/MS score”,

1 $$\begin{aligned} \textrm{score} = \frac{n_{\textrm{interpreted}}}{{|N |}} \end{aligned}$$

for each parent candidate formula, where $n_{\textrm{interpreted}}$ is the number of mass peaks which are explainable as subformula of the putative formula for the unknown parent and $|N |$ is the total number of mass peaks in the spectrum. The parent candidate formulae with a score that fails to meet a certain user-defined threshold are rejected. This filter was implemented into the MZMine2 software package [41], tested on a metabolomic dataset of 48 compounds from an extract of *Schizosaccharomyces pombe* cells, and obtained the correct formula in 79% of the cases, which was more successful than a similar method of Böcker and coworkers [13]. However, since several other complementary methods were used in conjunction to eliminate parent candidate formulae by both workers, and different data sets were used, it is difficult to conclude whether the MS/MS score performed better than the computation of fragment trees.

Similarly to the fragmentation graph of Böcker and coworkers, Guillevic and coworkers [42] defined a “pseudo-fragmentation graph” (see Fig. 2(a)), where the vertices correspond to all possible (parent and fragment) candidate formulae and edges correspond to a formula-subformula relationship, from which they define a “likelihood estimator” that they use for “non-target screening” (i.e. “quantifying unknown substances in a sample with little or no *a priori* knowledge”). Mixtures of predominantly halogen-containing gas molecules were analysed via GC-EI-MS. In the construction of the pseudo-fragmentation graph, a pruning step was included to eliminate “singleton” vertices with no parent or child, corresponding to either noise mass peaks or candidate formulae that did not correspond to the fragment represented by the measured mass. The set of “maximal fragments”, which are vertices possessing children but no parents in the pseudo-fragmentation graph then corresponds to the set of parent candidates for a given mass spectrum, if the molecular ion is present. After further incorporation of isotopic information, they then arrive at the most likely whole-spectrum annotation. The method was tested on 23 molecules of mass up to 338 Da, producing the correct parent ion formula in 80 % of the cases.

Finally, Xing and coworkers [43] recently developed the software package BUDDY, which performs formula identification for MS/MS spectra by what they refer to as “bottom-up formula interrogation”. Essentially, all fragment masses $M_i$, alongside the theoretical “neutral loss” $M_1 - M_i$ are used to query a formula database, yielding the query candidate subformula and candidate neutral loss set $C_i$ and $N_i$ respectively. The set of all possible (parent) candidate formula is then mathematically defined by the union

2 $$\begin{aligned} \bigcup ^{N_{\text {peak}}}_{i=1} \{c_{ij} + n_{ik} : (c_{ij}, n_{ik}) \in C_i \times N_i \} \end{aligned}$$

where $N_{\text {peak}}$ denotes the total number of mass peaks under consideration, and the addition term $c_{ij} + n_{ik}$ denotes the formation of new molecular formulae by adding the two formulae in compomer form. Informally, the assumption is that any valid candidate formula can be made by “stitching together” at least one fragment formula - neutral loss pair found within a molecular formula database, which is less restrictive and more amenable to discovering “unknown-unknowns” than directly searching the parent mass in the database.

Each candidate formula is then scored/ranked by a multiple linear regression (MLR) model, utilising 14 features derived from the isotopic profile of the precursor (MS$^1$) spectra and 24 features derived from the MS/MS spectra. The authors report superior performance than SIRIUS [40] on a number of datasets [43], however they also note that there is a some probability (which was small, based on their benchmarking datasets) of “misdisposition”, or the incorrect discarding of the correct formula, should it not lie within the set defined by Eq. 2, which is not the case with SIRIUS, since it generates all mathematically possible candidate formulae with a mass deviation below a certain threshold.

### This work

In this work, we outline a new method for whole-spectrum formula annotation, by first invoking the concept of what we call a the *subformula graph*, which represents a possible whole-spectrum formula annotation (i.e a mapping from the mass peaks in the spectrum to a corresponding candidate formula). This “subformula graph” is closely related to the “fragmentation graph” of Böcker and coworkers and defined similarly to that of the pseudo-fragmentation graph by Guillevic and coworkers, except we assume from the outset that there is a single dominant “parent” ion which fragmented to give rise to the majority of the fragments present in the mass spectrum. We propose this change in terminology to disambiguate the concept, because such a graph does not necessarily correspond to actual fragmentation events occurring within the mass spectrometer, or even the true formulae of the analyte and its fragments, instead simply encoding formula-subformula relationships of a number of putative formulae assigned to masses within the mass spectrum.

We further extend upon this concept by also defining the *parent subformula graph* (PSG), a specific type of subformula graph that is uniquely defined for each candidate parent formula with respect to a given mass spectrum. We show that the concept of the PSG is valuable, because subsequently each (candidate) parent formula can then be scored by some function depending on the topology of the PSG. To demonstrate this, we devised two simple scoring functions which solely depend on the connectivity of our PSG and do not invoke specific fragmentation rules or arbitrary data-dependent weights — a restriction that makes our method generally applicable across all subfields of mass spectrometry where fragment masses are present. We also show that given reasonably accurate mass measurements, should the correct parent formula be obtained, the corresponding PSG also corresponds to an (approximately) correct whole spectrum annotation.

Our method (construction of the PSG, followed by application of either of our scoring functions) has been benchmarked using hundreds of mass spectra (both EI-MS and MS/MS spectra) against our implementation of the existing “MS/MS score” of Pluskal and coworkers [14], corresponding to a vertex count of the PSG, as defined in this work^1^ and show that both of our scoring functions perform better than the “MS/MS score”. We also validate our method on “non-ideal” GC-EI-MS data, and show that in the case of EI-MS spectra where the identity of the molecular ion is unclear, a slight modification of our method can extract both the correct molecular ion (mass) and its corresponding molecular formula from the mass spectrum, even at trace levels, if it is present. Finally, we exhibit a way to represent the PSG; the two-dimensional fragmentation plot (2DFP), which is similar stylistically to plotted 2D-NMR data. We demonstrate the utility of these plots in determining whether or not a certain annotation may be reasonable, thus suggesting that candidate formula identification can be performed by visually inspecting the PSG, as a complement to using scoring functions.

**Table 1:** User-specified parameters and scoring function choices, along with their definitions

Parameter	Equations	Description
${\varvec{E}}, {\varvec{m}}, N$	(5)-(6)	The alphabet of elements, their masses, and their number
$\delta =\delta _1$	(10)	Threshold for likely parent-candidate formula selection
$\delta _{2\ldots N_{\text {peak}}}$	(24)	Threshold for likely fragment-candidate formula selection
$M_{\text {lower}}$, $M_{\text {upper}}$	(25)	The minimum and maximum plausible masses for parent ions
$I_{\text {noise}}$	(4)	Intensity cutoff for fragment masses
$s_{\text {ne}}$	(20)	Normalized edge-count score
$s_{\text {LBJ}}$	(22)	Li-Bohman-Jayatilaka product score
$s_{v}$	(18)	MZmine 2 normalised vertex count

## Methods

In this section we provide a mathematical description of our method for whole-spectrum annotation. We also relate our scheme to those employed by Pluskal and coworkers [14], and the fragmentation tree of Böcker and coworkers [36], and show how our scheme can be used to analyse mass spectra from weak samples with overlapping compounds. Table 1 summarizes the key parameters to be defined.

### Basic notation and terminology

We closely follow the notation of our earlier paper [44] and others [37, 42], and for convenience the defined terms are in italic. A mass spectrum is considered here to be a list of $N_{\text {peak}}$ *mass peaks*

3 $$\begin{aligned} {\varvec{M}} =\,&[ M_1, M_2, \ldots , M_{N_{\text {peak}}} ], \ {\text {where, for convenience,}} \nonumber \\&M_1> M_2> \ldots M_{N_{\text {peak}}}> 0, \ {\text {and}}\ M_{\text {max}} > M_1. \end{aligned}$$

$M_{\text {max}}$ is a maximum mass parameter that must be supplied in all analyses below, and ideally should be chosen as small as possible. The relative intensities associated with the mass peaks can be defined analogously,

4 $$\begin{aligned} {\varvec{I}} = [I_1, I_2 \ldots I_{N_{\text {peak}}}] \end{aligned}$$

where $I_i$ is the intensity associated with mass $M_i$. While most peaks are assumed to arise from a dominant *parent molecule* (of mass $M_1$) and its fragments, some may not be, and are regarded as interfering masses. Nevertheless, we refer to all non-parent masses as *fragment masses*. Following the notation of our previous paper [44], the unknown parent is assumed to be composed of elements in the *elemental list* or *alphabet*,

5 $$\begin{aligned} {\varvec{E}} = [ E_1, E_2, \ldots , E_N ], \end{aligned}$$

which have to be decided *a priori*; the associated (neutral-charged) list of *atomic masses* are

6 $$\begin{aligned} {\varvec{m}} = [ m_1, m_2, \ldots , m_N ]. \end{aligned}$$

We are only concerned here with the masses of the most abundant isotopes; however differing isotopes could be represented as distinct “elements” in the list. The true *molecular formula* associated with each molecular mass $M_i$, is

7 $$\begin{aligned} (E_1)_{f_{i1}} (E_2)_{f_{i2}} \ldots (E_N)_{f_{iN}}, \end{aligned}$$

where the stoichiometric coefficients are ${\varvec{f}}_i$, also called a *compomer* [45]. Thus the parent molecule associated with the largest-mass peak $M_1$ has the formula associated with the coefficients ${\varvec{f}}_1$. We often refer to ${\varvec{f}}_i$ *as* the true molecular formula associated with $M_i$ where no confusion may arise; and define ${\varvec{f}}$ to be the true *whole-spectrum formula annotation*. The *calculated* mass of the *i*-th peak is given by

8 $$\begin{aligned} M_i^{\text {calc}}&= M^{\text {calc}}({\varvec{f}}_i) = \sum _{k=1}^{N} f_{ik} m_k - Z m_e. \end{aligned}$$

$m_e$ is the electron mass, and *Z* the charge of the ion, which assuming the ion is singly charged is $+1$ or $-1$, depending on ion mode.

### Likely candidate formulae

In mass spectrometry the error in $M_i$ is given as a relative quantity e.g. a typical instrument may have an error of $10^{-5} M_i$, or 10 ppm. The meaning of this error from a statistical point of view is not clearly defined in the existing literature; for example, given an arbitrary number of mass measurements, how many would possess a measurement error exceeding this value? Regardless, due to the existence of a mass error, which increases with analyte mass, it is not always possible to determine ${\varvec{f}}_i$ from $M_i$ with certainty. However, one may at least define a list of *likely candidate formulae*, if it is further assumed that the experimental masses are normally distributed random variables centred on $M^{\text {calc}}_i$ and with standard deviation $\sigma _i$,

9 $$\begin{aligned} M_i \sim \mathcal {N} (M^{\text {calc}}_i, \sigma _i). \end{aligned}$$

Notably, we use the standard deviation of the measurement error, which is *not* equivalent to the mass error usually given for mass spectrometers, but has statistical meaning. The $c_i$-th candidate, described by a chemical formula $(E_1)_{n_{i1}[c_i]} \ldots (E_N)_{n_{iN}[c_i]}$, with stochiometric coefficients ${\varvec{n}}_i[c_i]$, is defined so that its calculated mass lies within $\delta$ standard deviations of the measured mass peak $M_i$ i.e.

10 $$\begin{aligned} |\Delta M_i^{c_i}|&= \big | M_i - M^{c_i}_i \big | \,\le \, \delta \, \sigma _i, \end{aligned}$$

11 $$\begin{aligned} M_i^{c_i}&= M^{\text {calc}}({\varvec{n}}_i[c_i]), \ \ {\text {for }} 1\le c_i \le N^{\text {cand}}_i, \end{aligned}$$

where $\Delta M_i^{c_i}$ is the signed *mass deviation* for candidate $c_i$ with formula ${\varvec{n}}_i[c_i]$ and mass $M^{c_i}_i$ and $N^{\text {cand}}_i$ is the total number of candidates for $M_i$. If an element does not appear in the chemical formula its corresponding stochiometric coefficient is zero. A set of formulae that satisfy equation (10) are said to lie within a $\delta$ *window*. In this work, as in ref. [42], we generally use $\delta = 3$, corresponding roughly to a 99.7% confidence interval, but different values of $\delta$ will lead to different sized candidate formula lists. In addition, for convenience, the candidate formulae are ordered with respect to the absolute mass deviation, i.e.

12 $$\begin{aligned} {\varvec{n}}_i[1]&,{} & {} \ {\varvec{n}}_i[2] ,{} & {} \ldots ,{} & {} \ {\varvec{n}}_i[N^{\text {cand}}_i] \ \ \ {\text {implies that}} \nonumber \\ |\Delta M^1_i|< & {} {}&\ |\Delta M^2_i|<{} & {} \ldots <{} & {} \ |\Delta M^{N^{\text {cand}}_i}_i|. \end{aligned}$$

Note that while ${\varvec{n}}_i[1]$ is the candidate formula with the smallest absolute mass deviation $|\Delta M^1_i|$, it may not be the best formula assignment with respect to the mass spectrum as a whole; we expand on this below. The construction of lists of all mathematically possible candidate formula containing some or all of the elements in the alphabet and lying within certain limits of the measured mass peak comprises a variant of the knapsack problem, known as the *money-changing problem* see e.g. [42, 44, 46], whose solutions for molecules of weight up to $M = 1000$ can be easily and efficiently read off from a *molecular formula tree* [44].

A problem that may arise in practice is the choice of $\sigma _i$ for an arbitrary mass spectrum. One possible way of obtaining this value is by empirically measuring the mass deviation of a large number of known compounds (and fragments) on the particular instrument, and then estimating $\sigma _i$ based on the aforementioned assumption of the error being normally distributed. If this cannot be done, then the experimentalist may choose to intentionally overestimate $\sigma _i$. While this introduces a number of additional candidate formulae, one can nonetheless be reasonably confident that the correct formula will be taken into account.

### The subformula restriction

We have assumed that most mass peaks in the spectrum arise from a dominant parent molecule, which is nothing other than assuming that separation and purification of the unknown has been (somewhat) successful. Assuming further that each fragment arises from a unimolecular dissociation reaction we are led to the *subformula restriction* [37], namely that

13 $$\begin{aligned} {\varvec{f}}_i&\preceq {\varvec{f}}_1, \ {\text {which means that}}\ f_{ik} \le f_{1k},\ {\text {for all}}\ 1 \le k \le N \end{aligned}$$

which is a formalisation of the assumption that the atoms in any fragment ion in the mass spectrum must be a subset of the atoms in the parent ion. The partial order symbol $\preceq$ can be read as “precedes or is equal to”, and it is transitive [36] i.e.

14 $$\begin{aligned} {\varvec{f}}_i&\preceq {\varvec{f}}_j \ {\text {and}}\ {\varvec{f}}_j \preceq {\varvec{f}}_k \ {\text {implies}}\ {\varvec{f}}_i \preceq {\varvec{f}}_k. \end{aligned}$$

As before, we extend the meaning of this symbol to the corresponding molecular formula e.g. for a hydrocarbon parent $\hbox {C}_{2}\hbox {H}_{2}$, we may write $\hbox {CH}_{2} \preceq \hbox {C}_{2}\hbox {H}_{2}$, which means $[1,2] \preceq [2,2]$ with respect to alphabet $[{\text {C}},{\text {H}}]$. However, $\hbox {C}_{4}\hbox {H}_{4} \npreceq \hbox {C}_{2}\hbox {H}_{2}$, or equivalently, $[4,4] \npreceq [2,2]$. Devising the subformula restriction allows us to define the *neutral loss formula* of ${\varvec{f}}_i$, with respect to ${\varvec{f}}_1$:

15 $$\begin{aligned} {\varvec{l}}_i = {\varvec{f}}_1 - {\varvec{f}}_i. \end{aligned}$$

Equation (13) then implies that $l_{ik} \ge 0$, for all $1 \le k \le N$, which means ${\varvec{l}}_i$ is always a valid molecular formula (with non-negative coefficients).

### The subformula graph

Aside from the subformula restriction, for a collection of fragments in a mass spectrum, one may also intuit that it is likely (but not guaranteed) that ${\varvec{f}}_i \preceq {\varvec{f}}_j$, for a given pair of fragment masses $M_i$ and $M_j$, where $M_j > M_i$. A convenient way to encode these relationships for a collection of molecular formulae present in a mass spectrum is via the *subformula graph* (SG), as illustrated in Fig. 2(b). Typically, a graph is defined by two sets; a set of $N_V$ *vertices*, or nodes, and a set of pairs of vertices, called *edges*. Since the SG is a (weakly) connected graph, due to the root vertex ${\varvec{f_1}}$ being connected to all other vertices, it can be defined as

16 $$\begin{aligned} {\text {SG}}({\varvec{f}})&= \bigg \{\, (V_i, V_j) \,\bigg |\, V_i \equiv {\varvec{f}}_i,\ V_j \equiv {\varvec{f}}_j, \ {\varvec{f}}_i\preceq {\varvec{f}}_j \,\bigg \} \end{aligned}$$

Each of the vertices $V_i$ represents a distinct annotated mass peak $M_i$ in the mass spectrum labeled by formula ${\varvec{f}}_i$, so in an SG we can refer vertices and mass peaks interchangeably.^2^ Note that the set of edges in the SG is entirely determined by the whole-spectrum formula annotation ${\varvec{F}}$, since if ${\varvec{f}}_i\preceq {\varvec{f}}_j$ then the pair of vertices $(V_i,V_j)$, or edge, must be in the SG set. We may also sometimes write $V_i \preceq V_j$, an abuse of notation, which implies a subformula restriction on the corresponding formula associated with the vertices, or mass peaks. It is important to note that not all mass peaks can be labelled by a (fragment) formula of the analyte. In these cases, if the assumption of unimolecular dissociation holds, the masses may have originated from instrumental or chemical noise. Thus, $N_V \le N_{\text {peak}}$ for any arbitrary SG.

### Subformula graphs made from candidate formula lists

Since there is uncertainty concerning the assignment of a molecular formula to a particular mass peak, we suggest that ranking whole-spectrum formula annotation may be better than assigning the formula with the smallest mass deviation to each mass separately. The first step in developing this idea is to define the set of all possible whole-spectrum assignments ${\varvec{n}}[{\varvec{c}}]$. Naively, this is just the set of candidates $\{ {\varvec{n}}_i[c_i] \}^{i=N{\text {peak}}}_{i=1}$ taken over all combinations of $c_1, c_2 \ldots c_{N_{\text {peak}}}$. This is evidently a very large number: if there were only two candidates per mass peak (and in practice there may be hundreds) the number would be $2^{N_{\text {peak}}}$. For our purpose, we consider this number impractically large. In order to navigate this massive search space, we first define the concept of a *valid* whole-spectrum annotation: ${\varvec{n}}[{\varvec{c}}]$ is valid iff $\forall i, \ {\varvec{n}}_i[c_i] \preceq {\varvec{n}}_1[c_1]$. In other words, all fragment candidate formulae must be subformulae of the parent candidate formula. This implies that for some ${\varvec{n}}_1[c_1]$, in order to compute a possible whole-spectrum annotation one only need to choose from a subset of all candidates $\{{\varvec{n}}_i[c_i]\}^{c_i = {N^{\text {cand}}_i}}_{c_i = 1}$; those which are subformulae of ${\varvec{n}}_1[c_1]$. This reduces the time required to generate feasible candidates, as the search space is reduced from all possible formulae to all possible subformulae of ${\varvec{n}}_1[c_1]$, which is much smaller. Also, due to the subformula constraint, we can encode any valid whole-spectrum assignment ${\varvec{n}}[{\varvec{c}}]$ as an SG, with each vertex instead associated with the corresponding candidate formula,

17 $$\begin{aligned} {\text {SG}}({\varvec{n}}[{{\varvec{c}}}])&= \bigg \{\, (V_i, V_j) \,\bigg |\, V_i \equiv {\varvec{n}}_i[c_i],\ V_j \equiv {\varvec{n}}_j[c_j],\ {\varvec{n}}_i[c_i]\preceq {\varvec{n}}_j[c_j] \,\bigg \}. \end{aligned}$$

Here ${\varvec{n}}[{\varvec{c}}]$ is a particular whole spectrum formula annotation defined for candidates $c_i$ in ${\varvec{c}}$, which has  size $N_{\text {peak}}$, but as before there are only $N_V[{\varvec{c}}]\le N_{\text {peak}}$ vertices in the SG, due to the subformula restriction.

### Number of vertices in the candidate subformula graph

A key observation that can be made is that the value of $N_V[{\varvec{c}}]$ for *any* given SG(${\varvec{n}}[{\varvec{c}}]$) depends only on the choice of the parent candidate ${\varvec{n}}_1[c_1]$, as the subformula restriction is applied with respect to the parent. By Occam’s razor, we can therefore conclude that a parent candidate which possess a higher $N_V[{\varvec{c}}]$ is a better (more parsimonious) “explanation” for the fragment mass than one with a lower $N_V[{\varvec{c}}]$, since we do not need to assume that many mass measurements either have an anomalously high mass deviation, or originate from noise. This singular principle underpins many of the existing methods for molecular formula annotation that uses fragment information, such as those of Pluskal and coworkers [14], Grange and coworkers [7], or Meringer and coworkers [37]. In the latter method, the criterion for an “annotated” fragment mass is the existence of a candidate fragment formula ${\varvec{n}}_i$ within some mass deviation of $M_i$, while in the former two methods, the criterion is instead the existence of a corresponding neutral loss ${\varvec{l}}_i$ which is a subformula of the parent, within some mass deviation of the mass difference $M_1 - M_i$. Since every ${\varvec{n}}_i$ satisfying the subformula restriction possess a valid ${\varvec{l}}_i$, the two methods are equivalent for a sufficiently large $\delta$, where the probability of an anomalous mass measurement possessing a deviation surpassing the window is negligible. The above naturally allows for a score $s_{v}({\varvec{n}}_1[c_1])$ to be computed for a given parent candidate formula, based on the normalised vertex count

18 $$\begin{aligned} s_{\text {v}}({\varvec{n}}_1[c_1]) = N_V[{\varvec{c}}])/N_{\text {peak}} \end{aligned}$$

with $N_V$ defined as before. This is equivalent to Eq. 1, which is the “MS/MS score” used by Pluskal and coworkers [14].

### Parent subformula graphs

Following on from the observation regarding the importance of the parent candidate formula, and to further reduce the number of SGs, since the search space may still be potentially intractable, we consider the *parent subformula graphs*,

19 $$\begin{aligned} {\text {PSG}}({\varvec{n}}_1[c_1])&= \bigg \{\, (V_i, V_j) \,\bigg |\, V_i \equiv {\varvec{n}}_i[s_1],\ V_i \preceq V_j \bigg \}. \end{aligned}$$

where ${\varvec{n}}_i[s_1]$ denotes, for some candidate formula ${\varvec{n}}_i[k]$, the smallest *k* such that ${\varvec{n}}_i[k] \preceq {\varvec{n}}_1[c_1]$. In other words, every fragment mass $M_i$ is annotated with the molecular formula possessing the *least* mass deviation, subject to the constraint that it is a subformula of ${\varvec{n}}_1[c_1]$. Clearly, there are only $N^{\text {cand}}_1$ graphs of this type, one for each parent candidate, meaning these SGs can be uniquely labelled by and thus used to score each ${\varvec{n}}_1[c_1]$. We also can define a *neutral loss parent-candidate subformula graph*, ${\text {NSG}}({\varvec{n}}_1[{c}_1])$, the counterpart to ${\text {PSG}}({\varvec{n}}_1[{c}_1])$, except that each vertex $V_i$ is labeled by the neutral-loss formula with least mass deviation (with respect to $M_1 - M_i$) ${\varvec{l}}_i[{s}_1]$. Our later results indicate that this corresponds a *worse* approximation of the correct fragment formula annotation than the PSG. Thus, all metrics (detailed in the next section) computed are calculated with respect to the PSG, as opposed to the NSG.

### Scoring parent-candidate subformula graphs

A number of possible ways of ranking different parent candidate formulae may be derived based on the PSG. They could be, for instance, based on the presence of specific edges in the PSG (neutral losses), or specific vertices (e.g weighting by the intensity of the masses corresponding to formulae annotations in the PSG) or some general topological feature of the PSG, which may also be parameterised empirically to optimise its performance in distinguishing between correct and incorrect formulae. However, to most clearly demonstrate that scoring metrics based on the PSG possess an advantage above and beyond the well-accepted and closely related “MS/MS score”, and to maximise interpretability of our method over performance, we instead define three different scoring functions based purely on the connectivity of the PSG, which are *not* parameterised with empirically derived weights and are arguably the simplest that can be conceived. The first is the *normalized edge count*

20 $$\begin{aligned} s_{\text {ne}}\, ({\varvec{n}}_1[c_1]) = \frac{2\big | {\text {PSG}}({\varvec{n}}_1[c_1]) \big |}{N_{\text {peak}}(N_{\text {peak}}-1)}, \end{aligned}$$

where $\big | {\text {PSG}}({\varvec{n}}_1[c_1]) \big |$ is the number of edges in ${\text {PSG}}({\varvec{n}}_1[c_1])$. The normalising factor $(N_{\text {peak}})(N_{\text {peak}}-1)/2$ represents the maximum number of (undirected) edges that could be entertained in a graph of $N_{\text {peak}}$ vertices; the theoretical maximum number of vertices in the PSG. This corresponds to using the number of child-parent subformula relationships as a measure of how well the spectrum is annotated as a whole. The next is the *graph density*

21 $$\begin{aligned} s_{\text {gd}}\,({\varvec{n}}_1[c_1])) = \frac{2\big | {\text {PSG}}({\varvec{n}}_1[c_1]) \big |}{N_V (N_V-1)} \end{aligned}$$

The normalising factor here is similar to before, but is instead based on a graph with $N_V\equiv N_V[c_1]$ vertices, the actual number of vertices in the PSG rather than the theoretical maximum. A shortcoming of this scoring function, which precludes its use, is that a subformula graph with only two vertices and one edge, which is likely incorrect, will have the maximal possible graph density score of 1. Since the normalised vertex count is small for this case, the graph density score may be corrected by using

22 $$\begin{aligned} s_{\text {LBJ}}\,({\varvec{n}}_1[c_1]) = s_{\text {v}}\,({\varvec{n}}_1[c_1])\,\, s_{\text {gd}}\,({\varvec{n}}_1[c_1]). \end{aligned}$$

Multiplying the graph density score with the vertex count (Eq. 18) penalises the overall score considerably in the just-mentioned deficient two-vertex one-edge case. Simultaneously, in cases where differing candidate formulae possess very similar or identical normalised vertex counts, incoporation of the graph density score allows for better discrimination between candidates. For convenience, we have labeled this score by the initials of the authors in this paper, but we also call it the *product score*. Regardless of the scoring function used, the actual optimum whole of spectrum formula assignment is obtained as one which maximises that score,

23 $$\begin{aligned} {\varvec{f}}&= {\mathop {\mathrm{arg\,max}}\limits _{c_1}}\ s_{\text {func}}\, ({\varvec{n}}_1[c_1]), \end{aligned}$$

where “func” is the chosen scoring function.

### Subformula graph versus Böcker and coworkers' fragmentation tree

We note that the “fragmentation tree”, or the maximum colourful subtree of Böcker and coworkers [36] can be readily related to the terminology of this work. For example, the “colourful” constraint corresponds to the annotation of each mass peak with a unique candidate formula. In our case, we circumvent the large search space of this problem by enforcing the heuristic of assigning each fragment mass to a subformula ${\varvec{n}}_i[S_1]$ with respect to a given candidate ${\varvec{n}}_1[c_1]$. Strictly speaking, this is only an approximation of the optimal fragment mass assignments. However, if the approximation is truly optimal, then the maximum colourful subtree with respect to ${\varvec{n}}_1[c_1]$ can simply be expressed as the maximum spanning tree within PSG(${\varvec{n}}_1[c_1]$), which is a much easier computational problem to solve than the NP-hard maximum colourful subtree problem (see [36]). Indeed, in the nomenclature of our work, the “brute force” algorithm for computing the maximum colourful subtree involves enumerating all possible (valid) SGs and finding the maximum spanning tree in each[36]. Given our interest is only the assignment of formulae to mass peaks and not a description of the hierarchy of fragmentation events in the mass spectrum, and like Guillevic and coworkers [42], we do not attempt to find this maximum spanning tree.

### Differing measurement precision for parent and fragment ions in noisy spectra

In mass spectra where the parent does not appear clearly (e.g. due to low concentration, high fragmentation or overlapping mass spectra of other compounds), one might be inclined to think there are more interfering masses. This suggests that a smaller value of $\delta$ should be used for fragment masses than for the parent,

24 $$\begin{aligned} \delta = \delta _1 > \delta _{2\ldots N_{\text {peak}}} \end{aligned}$$

The intuition is that this will reduce the likelihood that interfering masses are mistakenly given annotations in the subformula graph scoring procedure. This notion was tested in our results section.

### Finding the parent ion in noisy spectra

It may be the case that the largest mass $M_1$ in the spectrum is not the parent mass, due to interfering masses. In this case, we can locate the correct parent mass and then assign the correct parent formula, by widening the set of parent candidate formula,

25 $$\begin{aligned} {\varvec{f}}&= {\mathop {\mathrm{arg\,max}}\limits _{\begin{array}{c} p \in [N_{\textrm{upper}},N_{\textrm{lower}}] \\ c \in [1,N^{\text {cand}}_p] \end{array}}}\, s_{\text {method}}({\varvec{n}}_p[c_p]). \end{aligned}$$

Here $M_p$ is the mass of a potential parent, which we refer to as a “candidate mass”, since it is analogous to a candidate formula. It is assumed that the parent ion mass lies between an upper bound mass $M_{\text {upper}}$ and a lower bound mass $M_{\text {lower}}$. Therefore $N_{\text {lower}}$ and $N_{\text {upper}}$ correspond to indices of the smallest mass peak larger than $M_{\text {lower}}$ and smaller than $M_{\text {upper}}$ respectively. To eliminate potential interfering masses in noisy spectra, for each potential parent candidate of mass $M_p$, we create a filtered mass spectrum before the computation of the PSG. The *p*-filtered spectrum simply has all mass peaks $M_f < M_p$ with a base-peak normalized intensity $I_f$ below the threshold value $I_{\text {noise}}$ removed. Creating a separate *p*-filtered mass spectrum for each parent candidate mass $M_p$ prevents possible parent masses, which could potentially be of very low intensity, from being discarded, while at the same time allowing low intensity fragment masses (which are likely to be noise) to be filtered out. Of course, this method can work only if the molecular ion is detectible in the spectrum, a fact which might not be known *a priori*. Also, the method will fail if a potential parent has a formula that is a subformula of another potential candidate of a greater mass. In particular, it will be unable to distinguish a parent [M$_p$]$^+$ from adducts of it (e.g [M$_p$ + Na]$^+$).

### Benchmarking-choice of data sets

The three data sets below have been chosen to exhibit quite different characteristics: relatively large and well validated and isotopologue pruned MS$^2$, non-isotopologue pruned, and some spectra from GC-MS analysis of floral semiochemicals, taken from recently published studies. The latter provides a real example using crude solvent extracts of natural compounds separated firstly by GC and analysed by EI-MS to test how accurate the method is at determining the molecular formula from complex samples. All benchmarking datasets were compared with the vertex score $s_{\text {v}}$, corresponding to the “MS/MS” score.

#### CASMI 2016 competition: LC-MS/MS data

The Computational Assessment of Small Molecule Identification 2016 (CASMI 2016) data comprises 622 monoisotopic spectra [47]. This data set was curated as part of a blind competition held to benchmark the reliability of current computational methods for molecular structure determination from MS$^2$, with electrospray ionization being the soft ionization method used. It comprises mostly non-volatile metabolites, almost all analysed in positive ion mode. The data set contains molecules which may contain the 11 elements CHNOFSiPSClBrI. Formula annotations were available for the parent and fragment ions. These annotations were the best formula after some recalibration and “tie-breaking” procedures were used. For this data set, we assume $\sigma _i = 1$ ppm. We refer to the original publications for further details. Data was obtained from the MassBank database [48]. We also investigated whether our method could resolve binary mixtures of spectra from the CASMI-2016 dataset. Methological details, as well as the results this investigation are included in the Additional file 1.

#### RECETOX 2021 GC-EI-MS data: environmental pollutants

The RECETOX (Research Centre for Toxic Compounds in the Environment) exposome library of 386 high resolution GC-EI-Orbitrap MS spectra was curated for the purpose of identifying anthropogenic pollutant compounds, and contains compounds with a “broad physicochemical diversity and toxicological importance” [49]. The molecules in this library contain the 10 elements CHNOFSiPSClBr. Unlike CASMI 2016, since these are GC-EI-MS spectra, and no post-hoc de-isotoping was performed, isotopologue masses were present in the spectra. For this data set, we assume $\sigma _i =$ 3 ppm. Out of the 386 spectra, 240 contained a detectable monoisotopic molecular ion. In some cases, the monoisotopic (parent) mass may have been removed in the data cleaning process if the abundance was too low. Again unlike CASMI 2016, only the parent mass, rather than fragment masses, were annotated with the corresponding molecular formula. The data was downloaded from the supplementary material of the original reference.

#### ORCHID 2023 GC-EI-MS data: sexually deceptive West Australian orchid semiochemicals

The dataset of twenty compounds was obtained from previous studies [50–56] on the pollination chemistry of sexually deceptive West Australian orchids. Mass spectra were acquired using EI-TOFMS on a Waters GCT Premier TOF-MS connected to an Agilent 5975 GC equipped with a BPX5 [(5% phenyl polysilphenylene-siloxane), 30 m x 0.25 mm x 0.25 µm film thickness, SGE Australia] column, using helium as a carrier gas. The MS data is derived directly from biological extracts where target volatile compounds were separated by GC and is useful to test the method for determining molecular formulae of target compounds in the presence of potentially co-eluting background metabolites. In general, these samples contain very low amounts of the targeted compounds, in a complex sample matrix. For this data set, we assume $\sigma _i =$ 10 ppm and the elements CHNOS are present. We also set $M_{upper}$ and $M_{lower}$ to 250 and 130 Da respectively, as all compounds in this dataset lie within this mass range. We also show that each whole-spectrum annotation can be presented as a two-dimensional fragment plot (2DFP), which is analogous to graphical representations of 2D NMR data; see Fig. 6 for a more detailed explanation of the plots.

### Fragment formulae identification

The choice of whether or not to compute the PSG or NSG leads to potentially a different assignment of fragment formulae even for the same parent formula, since the formula with least mass deviation to $M_i$ is not necessarily the formula with least mass deviation to $M_1 - M_i$. This means that the performance of our method will differ depending on if the PSG or NSG is used. Thus, in order to assess whether computing the PSG or NSG leads to the best performance, we decided to directly assess the rate of identification of fragment formulae depending on whether the PSG or NSG is computed. Note that in the case of NSG computation, each vertex corresponds to a formula assignment to neutral loss $M_1 - M_i$ instead of an assignment to $M_i$. To convert this neutral loss formula back to a fragment formula ${\varvec{n}}_i[c_1]$ so we can check the correctness of the annotation, we simply take the (vector) difference ${\varvec{f}}_1 - {\varvec{l}}_1[1]$, where ${\varvec{f}}_1$ is the correct parent formula.

### Spectra removed and choice of element alphabets

Unless otherwise stated, we discarded all spectra that contained only the parent mass peak, since our method relies on the presence of fragmentation information. We also discarded spectra in which the parent mass peak is not present, or spectra where there is only one parent candidate formula. The number of spectra that remain for analysis then depends on the choice of alphabet. Following others [40] we sometimes use a fixed “base” alphabet comprising the elements CHNO and then any of the elements in PFSClBrI, if those were also present in these molecules. We denote this CHNO+. Likewise, CHNOP+ denotes a base alphabet of CHNOP and then any of the elements in FSClBrI, CHNOPF+ is the base alphabet of CHNOPF and any of the elements in SClBrI. Outside of benchmarking, in actual untargeted analysis, it is clearly not possible to know for certain beforehand which elements are present and which are not. However, we justify these alphabets in practice for the case of SClBrI, since the presence of these elements can be identified with high probability from the presence of strong isotopologue mass peaks. In the case of the elements F and P, those elements are rather less common for natural systems, and one may know beforehand that the sample may (or may not) contain compounds possessing these elements. Table 2 summarises the number of spectra in these categories, along with some results, which are discussed later.

### Validation metrics

To assess the number of correctly identified spectra we used the rank *r* of the correct parent molecular formula, based on the scoring method used (should two parent candidates possess identical scores, its mass deviation is used to break the tie), and the relative ranking position (RRP),

26 $$\begin{aligned} {\text {RRP}}&= \frac{r-1}{N_{\text {cand}}-1}. \end{aligned}$$

We report averages of the RRP value, and also provide a histogram of average RRP as a function of number of likely candidates.

The correctness of our whole-spectrum (i.e fragment formulae, not just parent formula) annotation for the CASMI 2016 data set was measured using the *true positive rate*,

27 $$\begin{aligned} {\text {TPR}}&= \frac{{\text {No. of formula matching the reference annotation}}}{{\text {Total number of reference annotations}}}, \end{aligned}$$

and the *positive predictive value*,

28 $$\begin{aligned} {\text {PPV}}&= \frac{{\text {No. of formula matching the reference annotation}}}{{\text {Total number of annotations by our method}}}. \end{aligned}$$

We decided not to use the *true negative rate*, since our method can either incorrectly identify a noise peak with a formula, or correctly identify a fragment peak, but annotate it with the wrong formula. The TPR and PPV could not be used for the RECETOX 2021 library since fragment masses were not annotated. In tests for both the parent and fragment formula annotations, we define a “match” or positive hit as an exact formula match to the monoisotopic peak.

### Program and availability

A Python 3 program that implements the methods described in this paper and generates 2DFPs from an input mass spectrum is available at github.com/seanli9604/subformula_graph, alongside all data analysed and scripts used to perform data analysis. An archived version is available at 10.5281/zenodo.7937927. The program is split up over five modules. The formula module generates the molecular formulae needed for our method using the molecular formula tree method in our earlier work [44]. The spectrum_reader module parses mass spectra in a variety of file formats, and corrects for the electron mass (and proton masses in the case of MS/MS), the mass_spectrum module computes and scores the PSGs, and the visualisation module generates the 2-dimensional fragment plots. Finally, the constants module contains symbols, nominal masses, exact masses, and DBE of the elements CHNOFSiPSClBrI, which can be modified should the user wish to include custom elements and/or isotopes. The Python libraries networkx, matplotlib, seaborn, netCDF4, numpy and pandas are used in the program. Currently accepted file formats for mass spectra are CSVs, MassBank data files (.msp), JCAMP-DX, or netCDF (.cdf).

A key advantage of using the molecular formula tree method for mass decomposition is that the method allows for a list of molecular formula trees of all masses $m < M$, encoding all possible molecular formulae with nominal mass *m*, to be pre-computed [44]. This optimisation considerably improves the speed of our program, since our method requires $N_{\text {peak}} * N^{\text {cand}}_1$ mass decompositions to generate all whole-spectrum formula annotations with respect to one parent mass $M_1$. With this optimisation, it only took 464 s to analyse all mass spectra in the CASMI-2016 data set (CHNOPF+), 102 s for the RECETOX data set and 44 s for the ORCHID data set, suggesting that our program can be used for high-throughput applications.

In our analysis, it was assumed that the (neutralised) parent formula possesses an non-negative integer ring plus double bond (RDBE) parameter, all fragment formulae possess non-negative (but not necessarily integral) RDBE values, and that each parent candidate formula contained more than 25% carbon by mass.

### Details of the computer used

A standard Lenovo 81GC laptop with 8Gb of physical memory installed, an Intel(R) Core(TM) i7-8550U CPU, 4 cores, 8 logical processors, and a clock speed of 1.8GHz was used for all calculations.

## Results and discussion

### Fragment formula annotation

**Fig. 3:**
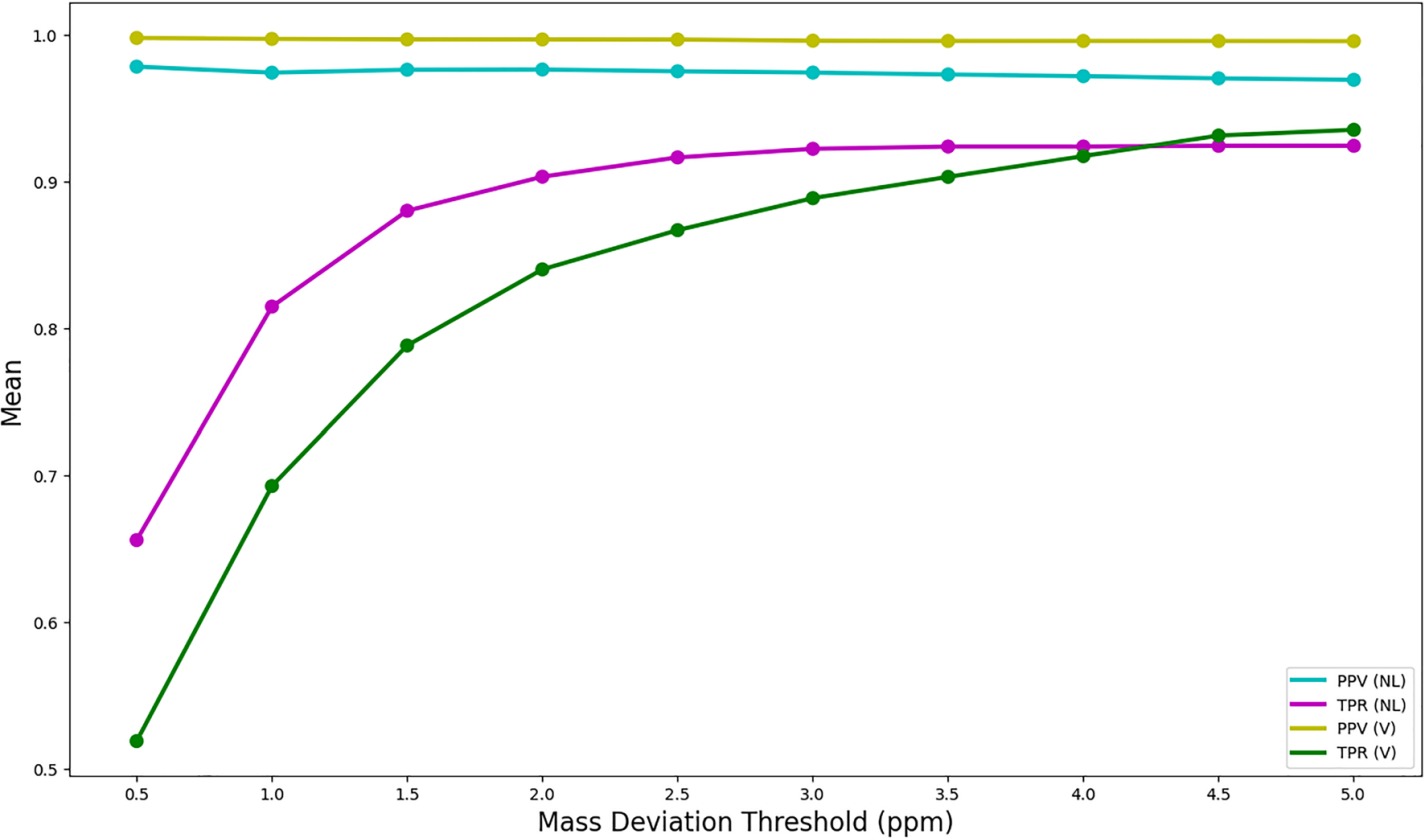
The mean PPV and TPR obtained for the fragment annotations, computed via constructing the PSG (denoted by “V”) and the NSG (denoted by “NL”) as a function of the mass deviation cutoff ($\sigma _i$), obtained by applying our method to all 622 compounds in the CASMI 2016 dataset

The results on the mean true positive rate (TPR) and positive predictive value (PPV) for fragment formulae identification is shown below in Fig. 3. In both the PSG and NSG case, TPR rapidly increased while PPV slowly decreased as the mass deviation limit increased. The rapid increase of the TPR as mass deviation increases is consistent with the increased proportion of fragment formulae that can be assigned a formula as the mass deviation increases. However, the PSG resulted in a notably higher number of correct fragment formulae annotations, with a mean PPV value of $\approx$ 0.996 even at the mass deviation cutoff of 5 ppm, in contrast to the 0.970 PPV of the NSG at the same mass deviation value. On the other hand, the TPR in the PSG case is generally lower than the TPR of the NSG case. However, past the mass deviation cutoff of 4ppm, the TPR of PSG becomes greater. The TPR of both methods plateau as the mass deviation cutoff increases further, due to the in-built restriction that any fragment formula candidates need to possess an RDBE value of zero or above.

These results suggest that for a given mass deviation cutoff, the NSG will successfully annotate more fragments in the mass spectrum, but for any given annotated fragment formula, computing the PSG is more likely to result in the correct annotation. For our purposes, we choose to compute the PSG as a means for annotating fragment formulae (and evaluating rankings), contrary to existing methods, which often rely on performing mass decompositions directly on mass differences. In addition, the above also suggests that, at least for mass deviations close to $\sigma _i = 1 \ \textrm{ppm}$, the fragment formulae assigned by the PSG heuristic are correct in the vast majority of cases, if the parent candidate formula is correct.

**Table 2:** Overall percentage success rates for predicting the rank of the correct parent formula ($r = 1$, $r \le 2$, or $r \le 4$) for the different data sets, for different alphabets, and for the different scoring functions. The average relative ranking position $\left\langle {\text {RRP}} \right\rangle$ and the average number of mass peaks $\left\langle N_{\text {peak}} \right\rangle$ is also reported. For the RECETOX data set, -ClBr indicates removal of molecules with more than three of these atoms, due to potential isotope peak interference

% Success rate per								
Data set	Element	Rank *r*	Scoring function			No. of	Total no.	$\left\langle N_{\text {peak}} \right\rangle$
	Alphabet	$\left\langle {\text {RRP}} \right\rangle$	$s_{\text {ne}}$	$s_{\text {LBJ}}$	$s_{\text {v}}$	Spectra	Of spectra	
CASMI	CHNO+	1	90.3	87.7	86.6	236	575	24.8
		$\le 2$	93.6	93.2	91.9			
		$\le 4$	97.0	97.5	95.3			
		$\left\langle {\text {RRP}} \right\rangle$	0.016	0.020	0.027			
	CHNOP+	1	80.0	82.4	72.1	365		
		$\le 2$	89.9	91.2	84.7			
		$\le 4$	94.2	94.8	91.2			
		$\left\langle {\text {RRP}} \right\rangle$	0.050	0.042	0.073			
	CHNOPF+	1	71.2	76.3	58.9	438		
		$\le 2$	82.6	87.0	73.5			
		$\le 4$	90.0	92.0	83.8			
		$\left\langle {\text {RRP}} \right\rangle$	0.058	0.046	0.091			
RECETOX	CHNO+	1	66.2	64.7	59.4	133	240	56.4
		$\le 2$	80.1	80.1	76.4			
		$\le 4$	87.0	90.1	87.8			
		$\left\langle {\text {RRP}} \right\rangle$	0.19	0.19	0.20			
	CHNO+ -3ClBr	1	77.5	75.5	68.1	94		45.1
		$\le 2$	87.0	87.0	81.5			
		$\le 4$	89.1	93.5	90.2			
		$\left\langle {\text {RRP}} \right\rangle$	0.09	0.09	0.11			
ORCHID	CHNOS	1	30.4	47.8	13.0	23	23	141.0
		$\le 2$	47.8	56.5	26.1			
		$\le 4$	56.5	87.0	30.4			
		$\left\langle {\text {RRP}} \right\rangle$	0.024	0.055	0.096			

### Overall performance of the formula annotation methods

Table 2 shows the performance of our scoring functions, in relation to the vertex score $s_{\text {v}}$, in terms of obtaining $r = 1$, $r \le 2$, and $r \le 4$ for the correct parent formula.

- On the CASMI data set, our two scoring functions and the (normalised) vertex score performed similarly with the smallest alphabet, about 86.6$-$90.3% success rate in obtaining $r=1$. We note a slight increase in the success rate of all categories, and a decrease in the mean RRP, for both of our scoring functions compared to the vertex score We expect a lower success rate with larger alphabet size, since there are more available candidates, and this is indeed observed; in this case the advantage of the normalized-edge and LBJ product scores becomes apparent over the normalised vertex count score. Also, as we relax the constraint from $r=1$, all methods perform better: the LBJ product score has the best success rate for obtaining $r \le 4$, and also for all but the smallest alphabet, in obtaining $r \le 2$, a fact that is also borne out in the averaged relative rank positions, $\left\langle {\text {RRP}} \right\rangle$.
- On the other hand, for the RECETOX data set over the alphabet CHNO+, the performance for obtaining $r=1$ is lower at 58.4$-$66.2% for all scoring functions; but, interestingly, the mean RRPs are not as high as might have been predicted from these success rates. Indeed, looking at how many formulae possess $r \le 2$, and $r \le 4$, we see a rapid rise, up to 90.1% for the LBJ product score function with $r \le 4$. Although a part of the decreased performance of all three methods may be attributed to the estimated experimental error, $\sigma = 3 \ {\text {ppm}}$, further investigation showed that the poor success rate for the correct formula in first place was due interference from isotopologue mass peaks in the spectrum. This was shown by removing all compounds with three or more chlorine or bromine atoms from the data set, and noting that the success rate increased significantly, with the normalised edge scoring function performing the best at 77.5% of correct parent formulae obtaining $r \le 1$ and the mean RRPs also halving. This demonstrates that our method are a direct complement to isotopic distribution-based methods; being significantly more effective in the case of mass spectra containing less isotopic information, as in the CASMI data set.
- For completeness, we consider the ORCHID data set, even though it was not chosen for validating formula annotation for a single, known parent mass. Nevertheless, we see an extremely poor performance from the vertex count score; it was only 30.4% accurate even in ranking the correct formula within the top four candidates. We conclude that the vertex or “MS/MS score” is not viable for locating the molecular ion and its corresponding formula in practice. What is very surprising is that the correct formula obtained $r \le 4$ for the LBJ scoring function in 87.0% of the spectra; recall that for these spectra from compounds in low amounts as parts of complex mixtures, *all* mass peaks greater than 130 Da were considered potential parents.

**Fig. 4:**
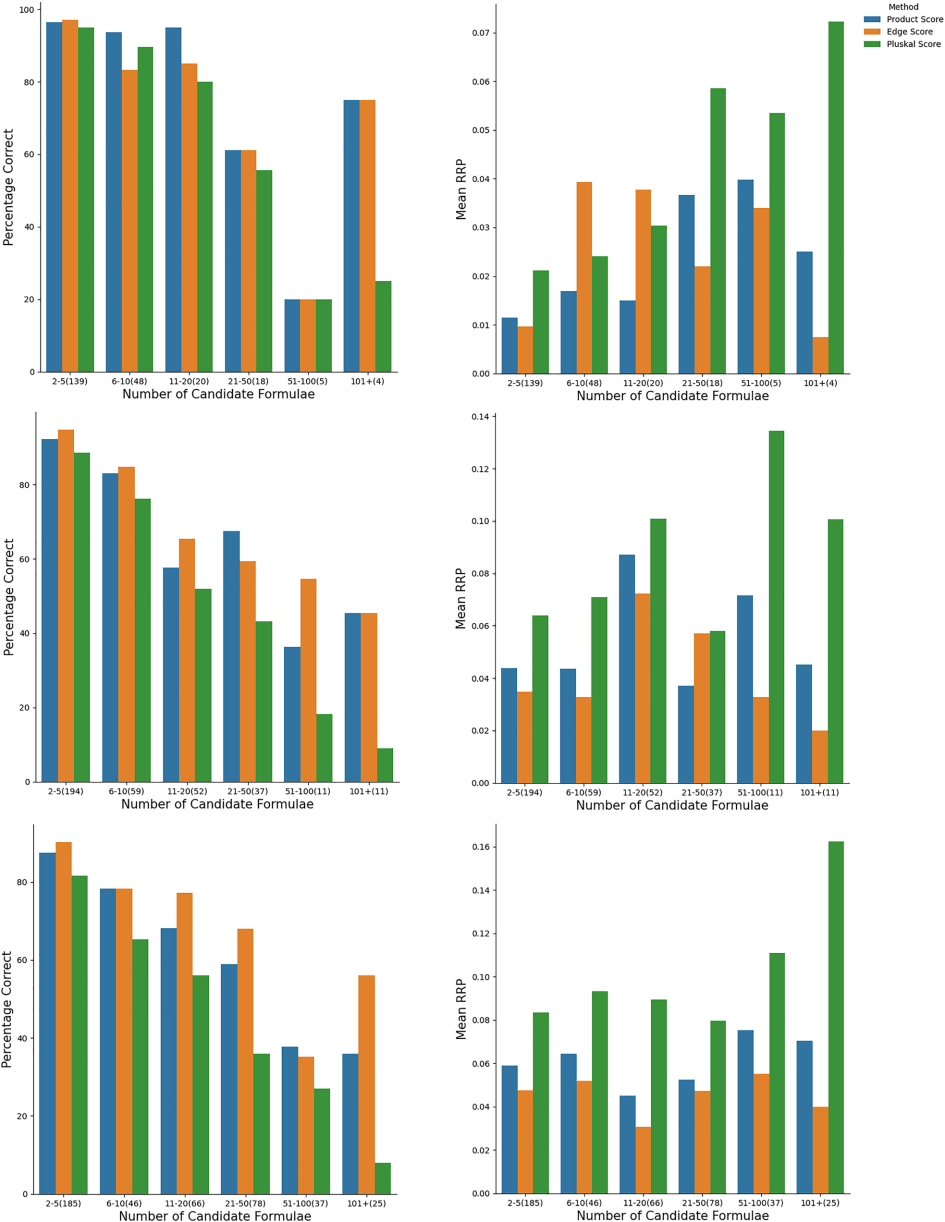
The mean success rate of obtaining $r = 1$ for the correct parent formula using the scoring functions $s_{\text {ne}}$, $s_{\text {LBJ}}$, and $s_{\text {v}}$ as a function of the number of likely parent candidates formulae $N_{\text {cand}}$ (left) and mean RRP (right), for the CASMI data using the alphabets CHNO+ (top), CHNOP+ (middle) and CHNOPF+ (bottom). The number of spectra in each bin is given in parentheses

### Dependence of success rate on the number of candidates

**Fig. 5:**
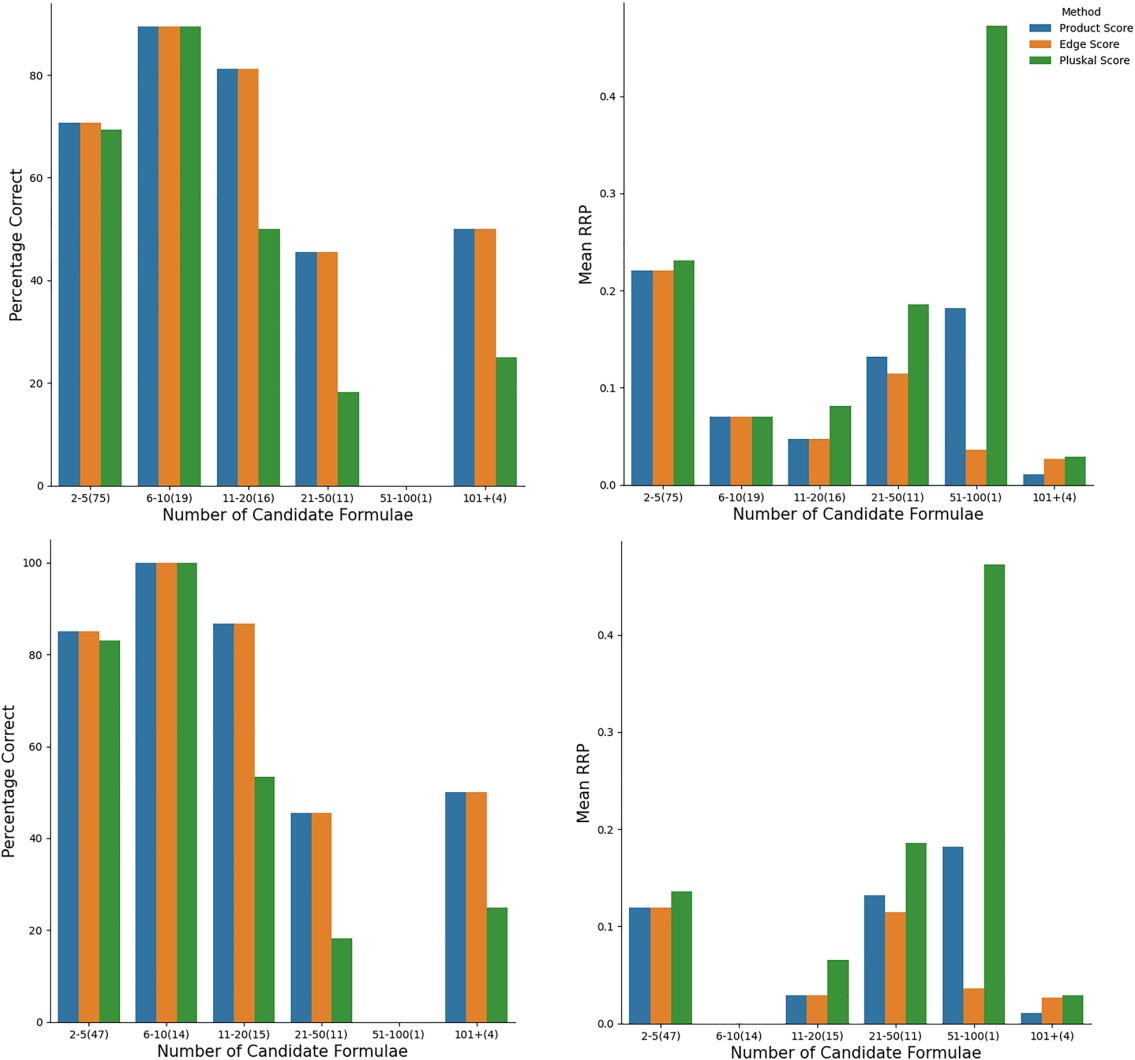
Breakdown of the rate of obtaining $r=1$ for the correct parent formula (left) and RRP (right) for the entire RECETOX dataset (top) and the same dataset with compounds possessing three or more chlorine or bromine atoms removed (bottom)

Figure 4 shows that there is a slightly better performance for $s_{\text {ne}}$ and $s_{\text {LBJ}}$ over the $s_{\text {v}}$ scoring functions; at least one of the two scoring functions $s_{\text {ne}}$ and $s_{\text {LBJ}}$ performs better than $s_{\text {v}}$ across all categories. We observe a general decrease in the success rate as the number of candidate formulae increases (correlated with the number of elements in the molecule). Overall, $s_{\text {LBJ}}$ performed slightly worse in correctly identifying formulae than the $s_{\text {ne}}$ for the CHNO+ element inclusion list. However, this scoring function consistently outperformed other scoring functions for the other two element inclusion lists, especially for large numbers of candidate formulae. On the other hand, Fig. 5 shows that in the case of the RECETOX dataset, the three methods performed much more similarly, with again a slightly better performance for $s_{\text {ne}}$ and $s_{\text {LBJ}}$ over the $s_{\text {v}}$ scoring functions, but only for the test cases with a relatively large ($> 10$) number of candidate formulae. In the other cases the performance was virtually identical. Overall, the results suggest that the increased ability (compared to $s_{\text {v}}$ for our scoring functions $s_{\text {ne}}$ and $s_{\text {LBJ}}$ to discriminate between correct and incorrect parent formulae is most apparent when there is a large number of candidate formulae (e.g larger parent masses, larger alphabet).

### Formula annotation for the ORCHID data in more detail

Overall, the molecular formula was correctly identified in 11 out of 23 cases with $s_{\text {LBJ}}$, and 7 out of 23 cases with $s_{\text {ne}}$, in comparison to just 3 out of 23 cases with $s_{\text {ne}}$. The correct molecular formula was in the top 4 ranked candidates, which is a reasonable number of candidates to examine and prune further manually, in 20 out of 23 cases with $s_{\text {LBJ}}$ and 13 out of 23 cases with $s_{\text {ne}}$, compared to 7 out of 23 cases with $s_{\text {v}}$. All test cases along with their ranks can be found in Additional file 1: Table S1 . In all cases, $s_{\text {LBJ}}$ produced better rankings for the correct formula than either $s_{\text {ne}}$ or $s_{\text {v}}$.

Due to the energy of bombarding electrons, it is possible that no trace of the intact molecular ion remains following ionisation. Indeed, this was the case with Compound 18; in this case, we decided that the “correct formula” is instead the largest fragment ion of the compound. However, in all other compounds in this data set, it was possible to identify the molecular ion and the corresponding candidate formula, even if the intensity is extremely low, and may be ordinarily discarded as “background noise” in the de-noising process or during mass spectral interpretation. This result brings into question the missing molecular ions in EI-MS. Certainly, in some cases the ion may be truly absent (or below the detection limit of the instrument), but many other cases where one may report the molecular ion as absent, perhaps the molecular ion was still present, in trace amounts.

In some cases, the correct formula is ranked lower than a “similar” formula, containing one or two extra hydrogens (e.g Compound 7, refer to Additional file 1: Table S8 ). This is likely due to $M + 1$ or $M + 2$ isotopologue peaks that were mistakenly assigned a formula, and would not pose a problem in applications where the mass spectrum undergoes de-isotoping and/or if a minimum intensity cutoff was also set for possible parent masses.

In this dataset, there are two notable test cases; Compound 15(a) and Compound 20, where the correct formulae were ranked very poorly by all three methods. In the case of Compound 15, the poor ranking may be attributed to the presence of a co-eluting compound. Raising the intensity cutoff to $I_{noise} = 0.05$ improves the rank of the correct formula drastically to fourth place with $s_{\text {LBJ}}$. In the case of compound 20, its molecular mass is very small ($M = 130$), so it is more likely for interfering ions that are significantly larger to be assigned formulae that overlap with the molecular formula of interest (i.e the correct formula is a subformula of the proposed candidate). Indeed, if we eliminate all formulae with $M > 200$, the correct formula $\hbox {C}_{6}\hbox {H}_{10}\hbox {O}_{3}$ is now the top ranked formula with $s_{\text {LBJ}}$ (Additional file 1: Table S22).

In general, the method seems quite effective in terms of locating a likely molecular ion and molecular formula for the most abundant compound contributing to a given EI-MS spectrum. Furthermore, in the cases where the identity of a compound remains ambiguous, there exists extra information within the PSG, which can be used to discriminate between candidates; namely, the mass difference $M_1 - M_2$ and the sum of intensities of assigned fragment masses.

For example, an indication that a large, erroneous mass is proposed as the molecular ion is if the value of $M_1 - M_2$ – the difference between the putative molecular ion and the largest mass annotated with a fragment formula, is very large. In chemical terms, this means that the smallest possible neutral loss has a high mass. The assignment is especially dubious if the corresponding neutral loss also consists of multiple heteroatoms. Naturally, there may be exceptions where a large $M_1 - M_2$ value is chemically sensible. Thus, we do not incorporate this into the ranking scheme; the value of $M_1 - M_2$ is instead displayed explicitly in the 2DFP.

Another indication of an incorrectly identified candidate formula is if the set of assigned molecular formulae and fragment formulae possesses a high score, but a number of peaks with high intensity cannot be assigned a fragment formula. In the case of a very small amount of compound this is not a reliable indication, since background or co-eluting compounds may result in relatively high intensity interfering mass fragments. However, if the peak is both sufficiently large and not overlapping, then this indicator would suggest that the molecular formula assignment is not appropriate. A detailed walkthrough of how these indicators can be used to identify the correct formula from a collection of likely candidate formulae (as obtained ranked by our method), with regards to specific cases within the ORCHID data set, is included in Section 3 of the Additional file 1.

**Fig. 6:**
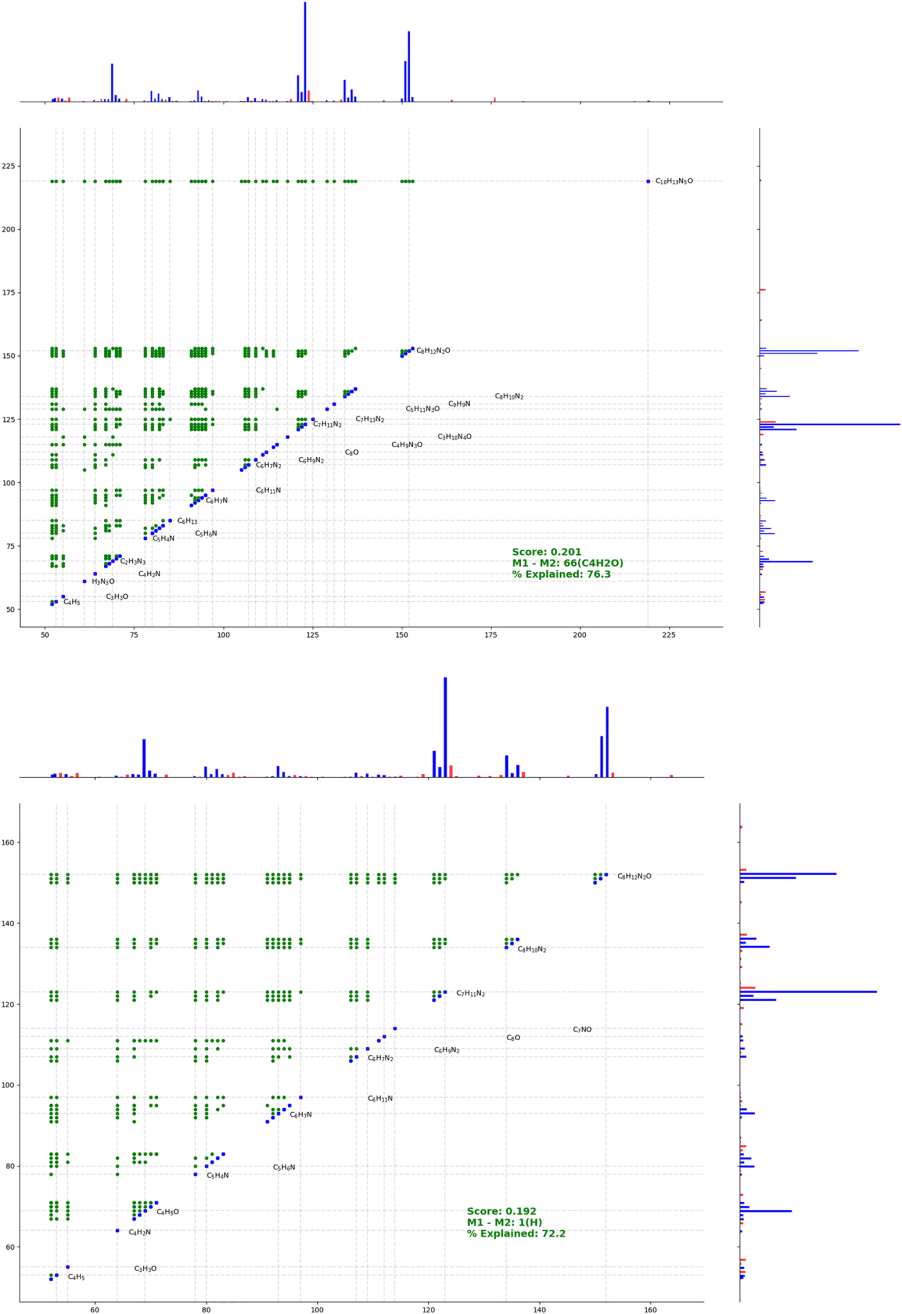
2DFP generated from the PSG derived from the highest scoring (incorrect) parent candidate formula, $\hbox {C}_{10}\hbox {H}_{13}\hbox {N}_{5}\hbox {O}$ (top) and from the correct parent candidate formula, $\hbox {C}_{8}\hbox {H}_{12}\hbox {N}_{2}\hbox {O}$ (bottom), for Compound 1. The masses in the mass spectrum are coloured either blue or red, signifying the presence or absence of a suitable (fragment) formula annotation for the peak respectively. Each green dot denotes a pair of “explained” masses such that the annotated formula of one is a subformula of another

### Discussion and future work

In this section we will discuss some possible improvements or further applications of our method.

#### Alternative scoring

A very clear point of improvement that can be made is in our scoring function for candidate formulae; our two scores directly encode the assumption that fragment formulae are likely to be subformulae of each other, and so is a proof-of-concept that this fact alone allows for the correct annotation to be distinguished from incorrect ones. However, clearly our method is unlikely to perform optimally with respect to any given data set. For example, a straightforward avenue for improvement is to weight the two components of $s_{\text {LBJ}}$ differently, for example

29 $$\begin{aligned} s_{\text {weighted}} = s_{\text {v}}({\varvec{n}}_1[c_1])^w s_{\text {gd}}({\varvec{n_1}}[c_1]) \end{aligned}$$

where *w* is some weighting factor that can be tuned for different classes of compounds, or different quality of data. Another way of improving our method is to utilise some other metric calculable from the SG, for example the length of the longest path or the size of the maximal (directed) clique in the SG. This could perhaps be utilised as parameters in an MLR model, analogous to what was implemented in the software package BUDDY by Xing and coworkers [43]. A third avenue of improvement for our method would be by incorporating (non-unit) weights into the edges of the SG, corresponding to rewarding certain (theoretical) neutral losses that are “logical” and penalising those that are “illogical”, perhaps with a machine learning process to acquire optimal weights from data. These avenues were not explored in this work, because we wish to first open up the possibility of developing methods for formula identification via the topology of networks constructed by formula-subformula relationships, such as the PSG in this work. Consequently, we believe it is best to first present the simplest, most interpretable scoring scheme before any additional complexity is introduced, to facilitate further development and/or implementation of similar methods and so that the advantages/disadvantages and the general nature of this class of methods are not obscured by implementation or training details.

#### Incorporation of isotopic information

Another way of improving our method in a straightforward manner would be to incorporate isotopic information into the score of a given parent candidate formula, which was done in ALPINAC, the software of Guillevic and coworkers [42] or MZMine 2/3 [14]. Aside from matching a simulated isotopic profile to the experimentally measured profile in the mass spectrum, our method can also be used to construct a PSG that include isotopologues, instead of only monoisotopic formulae. However, one would need to place appropriate restrictions on the maximum number/proportion of minor isotopic atoms for a given formula which would enable it to contribute significantly to the presence of some mass peak in the spectrum. Furthermore, it would also be necessary to build into the scoring function some way of taking into account the intensity of the mass peaks. As our main goal in this work is to propose and validate a new metric of evaluating whole-spectrum formula annotations, rather than create a comprehensive software tool for the analysis of mass spectra, we do not explore this option here.

## Conclusion

In this work, we clearly defined the concept of a subformula graph, previously existing within the literature as a component of other methods, in slightly different forms and under various other names. The subformula graph represents formula-subformula relationships amongst parent and fragment formulae. It is not only defined with respect to true parent/fragment formulae, but can also be constructed from putative (candidate) formulae annotations of a mass spectrum, and used to assess the validity of such an annotation.

We then show that, with respect to a particular parent candidate formula, we can construct a unique whole-spectrum annotation, represented as a type of subformula graph, the Parent Subformula Graph (PSG). We then devised two scoring functions, both of which incorporate the network density of the PSG, which can be used to assess the appropriateness of parent candidate formulae based on the whole-spectrum annotation derived from it.

We implemented this method into a Python program, and validated it on the CASMI-2016 (MS/MS) and Recetox (EI-HRMS) datasets. We first show that should the correct formula be obtained, the annotation of the corresponding fragment ions are also mostly accurate, indicating that, for high resolution instruments, the PSG is generally a good approximation of the subformula graph constructed from the true parent and fragment formulae.

Then, we demonstrate that our scoring functions consistently outperformed a simple vertex count (the MS/MS score, commonly used in existing software packages such as MZMine as a filter for parent candidate formula) at ranking the correct molecular formula favourably, especially in the case of large numbers of candidate formulae, in the case of both EI-MS and MS/MS spectra.

We also demonstrate with a smaller set of (GC-)EI-MS orchid semiochemical data that not only can our method be applied to “non-ideal” mass spectra possessing significant interfering masses, by repeatedly applying PSG construction and candidate scoring to all masses between a certain threshold value for plausible molecular ions, we can also detect the correct molecular mass (should such a mass be available, even at very low intensities) and correspondingly retrieve the correct molecular formula, as well as the whole-spectrum annotation.

In the case that multiple high-scoring candidate masses and/or formulae are obtained in the above method, we can represent the PSG generated as a 2D Fragment Plot (2DFP), which allows the analyst to investigate the likelihood or the validity of a number of possible parent candidate formulae more easily than directly interpreting the mass spectra. This shows that our method can be used to assist experimentalists in mass spectral interpretation, in addition to simply providing a score or ranking for candidates. The fact that spuriously high scoring formulae can be seen by examining the 2DFP also indicates the possibility of incorporating other features from the PSG into statistical models or machine learning methods for candidate formula identification, in order to boost the performance of the “naïve” method in this work.

### Supplementary information

**Additional file 1.**  Supplementary information. 

## Data Availability

The Python code implementing our method is available at github.com/seanli9604/subformula_graph, alongside all data analysed and scripts used to perform data analysis. An archived version of this repository is available at 10.5281/zenodo.7937927.

## Footnotes

1 We also have shown in our work, by benchmarking our method against it, that the “MS/MS score” can be applied to EI-MS data without issue.

2 In the mathematical literature, the definition of a graph also includes its vertex set; here, in the interests of brevity and readability, we leave these vertices defined implicitly.

## References

[1] Abate S, Ahn YG, Kind T, Cataldi TRI, Fiehn O (2010). Determination of elemental compositions by gas chromatography/time-of-flight mass spectrometry using chemical and electron ionization. Rapid Commun Mass Spectrom.

[2] McLafferty FW (1981). Tandem mass spectrometry. Science.

[3] Yost RA, Fetterolf DD (1983). Tandem mass spectrometry (ms/ms) instrumentation. Mass Spectrom Rev.

[4] Savitzky A, Golay MJE (1964). Smoothing and differentiation of data by simplified least squares procedures. Anal Chem.

[5] Gu H, Gowda GAN, Neto FC, Opp MR, Raftery D (2013). Ramsy: ratio analysis of mass spectrometry to improve compound identification. Anal Chem.

[6] Navarro-Huerta JA, Torres-Lapasió JR, Lóópez-Ureña S, García-Alvarez-Coque MC (2017). Assisted baseline subtraction in complex chromatograms using the beads algorithm. J Chromatogr A.

[7] Grange AH, Winnik W, Ferguson PL, Sovocool GW (2005). Using a triple-quadrupole mass spectrometer in accurate mass mode and an ion correlation program to identify compounds. Rapid Commun Mass Spectrom.

[8] Kind T, Fiehn O (2007). Seven golden rules for heuristic filtering of molecular formulas obtained by accurate mass spectrometry. BMC Bioinf.

[9] Yergey JA (1983). A general approach to calculating isotopic distributions for mass spectrometry. Int J Mass Spectr Ion Phys.

[10] Kubinyi H (1991). Calculation of isotope distributions in mass spectrometry. a trivial solution for a non-trivial problem. Anal Chim Acta.

[11] Sleno L, Volmer DA, Marshall AG (2005). Assigning product ions from complex ms/ms spectra: the importance of mass uncertainty and resolving power. J Am Soc Mass Spectrom.

[12] Stoll N, Schmidt E, Thurow K (2006). Isotope pattern evaluation for the reduction of elemental compositions assigned to high-resolution mass spectral data from electrospray ionization fourier transform ion cyclotron resonance mass spectrometry. J Am Soc Mass Spectrom.

[13] Böcker S, Letzel MC, Lipták Z, Pervukhin A (2009). Sirius: decomposing isotope patterns for metabolite identification. Bioinformatics.

[14] Pluskal T, Uehara T, Yanagida M (2012). Highly accurate chemical formula prediction tool utilizing high-resolution mass spectra, ms/ms fragmentation, heuristic rules, and isotope pattern matching. Anal Chem.

[15] Valkenborg D, Mertens I, Lemière F, Witters E, Burzykowski T (2012). The isotopic distribution conundrum. Mass Spectrom Rev.

[16] Wegner A, Weindl D, Jäger C, Sapcariu SC, Dong X, Stephanopoulos G, Hiller K (2014). Fragment formula calculator (ffc): determination of chemical formulas for fragment ions in mass spectrometric data. Anal Chem.

[17] Reemtsma T (2009). Determination of molecular formulas of natural organic matter molecules by (ultra-) high-resolution mass spectrometry: status and needs. J Chromatogr A.

[18] Tolic N, Liu Y, Liyu A, Shen Y, Tfaily MM, Kujawinski EB, Longnecker K, Kuo L-J, Robinson EW, Pasa-Tolic L, Hess NJ (2017). Formularity: software for automated formula assignment of natural and other organic matter from ultrahigh-resolution mass spectra. Anal Chem.

[19] Leefmann T, Frickenhaus S, Koch BP (2019). Ultramassexplorer: a browser-based application for the evaluation of high-resolution mass spectrometric data. Rapid Commun Mass Spectr.

[20] Schum SK, Brown LE, Mazzoleni LR (2020). Mfassignr: molecular formula assignment software for ultrahigh resolution mass spectrometry analysis of environmental complex mixtures. Environ Res.

[21] Leyva D, Jaffe R, Fernandez-Lima F (2020). Structural characterization of dissolved organic matter at the chemical formula level using tims-ft-icr ms/ms. Anal Chem.

[22] Wu QQ (1998). Multistage accurate mass spectrometry: a basket in a basket approach for structure elucidation and its application to a compound from combinatorial synthesis. Anal Chem.

[23] Konishi Y, Kiyota T, Draghici C, Gao J-M, Yeboah F, Acoca S, Jarussophon S, Purisima E (2007). Molecular formula analysis by an ms/ms/ms technique to expedite dereplication of natural products. Anal Chem.

[24] Rojas-Chertó M, Kasper PT, Willighagen EL, Vreeken RJ, Hankemeier T, Reijmers TH (2011). Elemental composition determination based on ms n. Bioinformatics.

[25] Scheubert K, Hufsky F, Rasche F, Böcker S (2011). Computing fragmentation trees from metabolite multiple mass spectrometry data. J Comput Biol.

[26] Kasper PT, Rojas-Chertó M, Mistrik R, Reijmers T, Hankemeier T, Vreeken RJ (2012). Fragmentation trees for the structural characterisation of metabolites. Rapid Commun Mass Spectr.

[27] McLafferty FW (1973) Interpretation of Mass Spectra, 2d ed., rev., enl., reset. edn. W. A. Benjamin, Reading, Mass

[28] Pellegrin V (1983). Molecular formulas of organic compounds: the nitrogen rule and degree of unsaturation. J Chem Educ.

[29] Senior JK (1951). Partitions and their representative graphs. Am J Math.

[30] Badertscher M, Bischofberger K, Munk ME, Pretsch E (2001). A novel formalism to characterize the degree of unsaturation of organic molecules. J Chem Inf Comput Sci.

[31] Kendrick E (1963). A mass scale based on ch2 = 14.0000 for high resolution mass spectrometry of organic compounds. Anal Chem.

[32] Hsu CS, Qian K, Chen YC (1992). An innovative approach to data analysis in hydrocarbon characterization by on-line liquid chromatography-mass spectrometry. Anal Chim Acta.

[33] van Krevelen DW (1950). Graphical-statistical method for the study of structure and reaction processes of coal. Fuel.

[34] Guo X, Bruins AP, Covey TR (2006). Characterization of typical chemical background interferences in atmospheric pressure ionization liquid chromatography-mass spectrometry. Rapid Commun Mass Spectrom.

[35] Schwarzenberg A, Ichou F, Cole RB, Machuron-Mandard X, Junot C, Lesage D, Tabet J-C (2013). Identification tree based on fragmentation rules for structure elucidation of organophosphorus esters by electrospray mass spectrometry. J Mass Spectrom.

[36] Böcker S, Rasche F (2008). Towards de novo identification of metabolites by analyzing tandem mass spectra. Bioinformatics.

[37] Meringer M, Reinker S, Zhang J, Muller A (2011). Ms/ms data improves automated determination of molecular formulas by mass spectrometry. MATCH Commun Math Comput Chem.

[38] Suzuki S, Ishii T, Yasuhara A, Sakai S (2005). Method for the elucidation of the elemental composition of low molecular mass chemicals using exact masses of product ions and neutral losses: application to environmental chemicals measured by liquid chromatography with hybrid quadrupole/time-of-flight mass spectrometry. Rapid Commun Mass Spectrom.

[39] Hufsky F, Rempt M, Rasche F, Pohnert G, Böcker S (2012). De novo analysis of electron impact mass spectra using fragmentation trees. Anal Chim Acta.

[40] Dührkop K, Fleischauer M, Ludwig M, Aksenov AA, Melnik AV, Meusel M, Dorrestein PC, Rousu J, Böcker S (2019). Sirius 4: a rapid tool for turning tandem mass spectra into metabolite structure information. Nat Methods.

[41] Pluskal T, Castillo S, Villar-Briones A, Oresic M (2010). Mzmine 2: modular framework for processing, visualizing, and analyzing mass spectrometry-based molecular profile data. BMC Bioinf.

[42] Guillevic M, Guillevic A, Vollmer MK, Schlauri P, Hill M, Emmenegger L, Reimann S (2021). Automated fragment formula annotation for electron ionisation, high resolution mass spectrometry: application to atmospheric measurements of halocarbons. J Cheminf.

[43] Xing S, Shen S, Xu B, Li X, Huan T (2023). Buddy: molecular formula discovery via bottom-up ms/ms interrogation. Nat Methods.

[44] Li S, Bohman B, Jayatilaka D (2022). Enumerating possible molecular formulae in mass spectrometry using a generating function based method. MATCH Commun Math Comput Chem.

[45] Böcker S, Lipták Z (2005) Efficient mass decomposition. In: Proceedings of the 2005 ACM Symposium on Applied Computing, pp. 151–157

[46] Bocker S, Liptak Z (2007). A fast and simple algorithm for the money changing problem. Algorithmica.

[47] Schymanski EL, Ruttkies C, Krauss M, Brouard C, Kind T, Dührkop K, Allen F, Vaniya A, Verdegem D, Böcker S, Rousu J, Shen H, Tsugawa H, Sajed T, Fiehn O, Ghesquiére B, Neumann S (2017). Critical assessment of small molecule identification 2016: automated methods. J Cheminform.

[48] Horai H, Arita M, Kanaya S, Nihei Y, Ikeda T, Suwa K, Ojima Y, Tanaka K, Tanaka S, Aoshima K, Oda Y, Kakazu Y, Kusano M, Tohge T, Matsuda F, Sawada Y, Hirai MY, Nakanishi H, Ikeda K, Akimoto N, Maoka T, Takahashi H, Ara T, Sakurai N, Suzuki H, Shibata D, Neumann S, Iida T, Tanaka K, Funatsu K, Matsuura F, Soga T, Taguchi R, Saito K, Nishioka T (2010). Massbank: a public repository for sharing mass spectral data for life sciences. J Mass Spectrom.

[49] Price EJ, Palat J, Coufalikova K, Kukucka P, Codling G, Vitale CM, Koudelka S, Klanova J (2021). Open, high-resolution ei+ spectral library of anthropogenic compounds. Front Public Health.

[50] Bohman B, Jeffares L, Flematti G, Phillips RD, Dixon KW, Peakall R, Barrow RA (2012). The discovery of 2-hydroxymethyl-3-(3-methylbutyl)-5-methylpyrazine: a semiochemical in orchid pollination. Org Lett.

[51] Bohman B, Phillips RD, Flematti GR, Barrow RA, Peakall R (2017). The spider orchid caladenia crebra produces sulfurous pheromone mimics to attract its male wasp pollinator. Angew Chem.

[52] Bohman B, Phillips RD, Menz MHM, Berntsson BW, Flematti GR, Barrow RA, Dixon KW, Peakall R (2014). Discovery of pyrazines as pollinator sex pheromones and orchid semiochemicals: implications for the evolution of sexual deception. New Phytol.

[53] Bohman B, Tan MMY, Phillips RD, Scaffidi A, Sobolev AN, Moggach SA, Flematti GR, Peakall R (2020). A specific blend of drakolide and hydroxymethylpyrazines: An unusual pollinator sexual attractant used by the endangered orchid drakaea micrantha. Angew Chem.

[54] Bohman B, Jeffares L, Flematti G, Byrne LT, Skelton BW, Phillips RD, W Kingsley Dixon, Peakall R, Barrow RA (2012). Discovery of tetrasubstituted pyrazines as semiochemicals in a sexually deceptive orchid. J Nat Prod.

[55] Bohman B, Weinstein AM, Phillips RD, Peakall R, Flematti GR (2019). 2-(tetrahydrofuran-2-yl)acetic acid and ester derivatives as long-range pollinator attractants in the sexually deceptive orchid cryptostylis ovata. J Nat Prod.

[56] Xu H, Bohman B, Wong DCJ, Rodriguez-Delgado C, Scaffidi A, Flematti GR, Phillips RD, Pichersky E, Peakall R (2017). Complex sexual deception in an orchid is achieved by co-opting two independent biosynthetic pathways for pollinator attraction. Curr Biol.

