# Supplementary material for: Determining the parent and associated fragment formulae in mass spectrometry via the parent subformula graph
Source: J Cheminform. 2023 Nov 7;15:104. doi: 10.1186/s13321-023-00776-y (PMC10631010; doi:10.1186/s13321-023-00776-y)
Supplement: Supplementary file 1 — Additional file 1. Supplementary information. [file 13321_2023_776_MOESM1_ESM.pdf]

Supplementary Information For "Determining the parent and associated fragment formulae in mass spectrometry via the parent subformula graph"

Sean Li, Björn Bohman, Gavin R. Flematti and Dylan Jayatilaka

September 22, 2023

1 CASMI-2016 Data Set

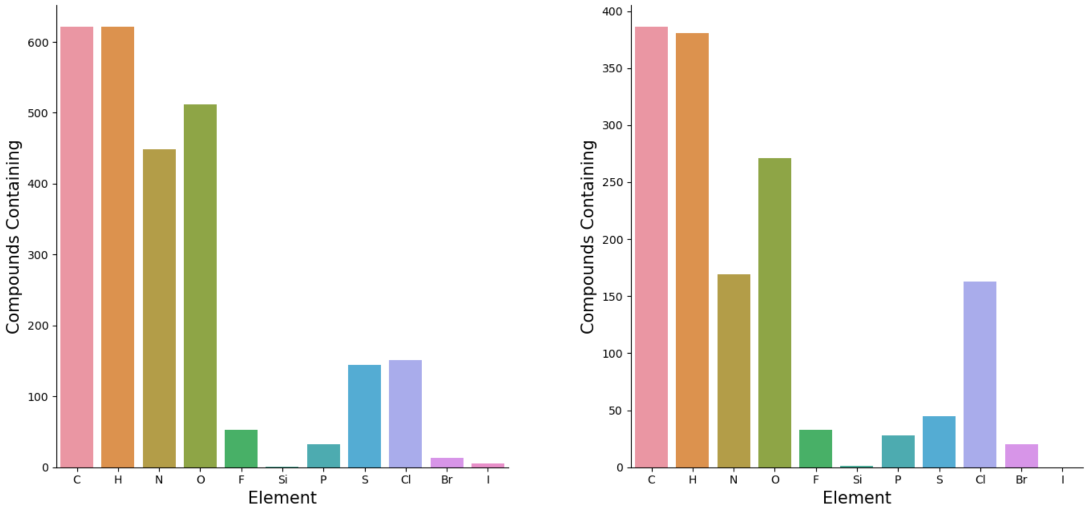

Figure 1: The number of test cases containing a given element in the CASMI-2016 dataset (left) and the Recetox dataset (right)

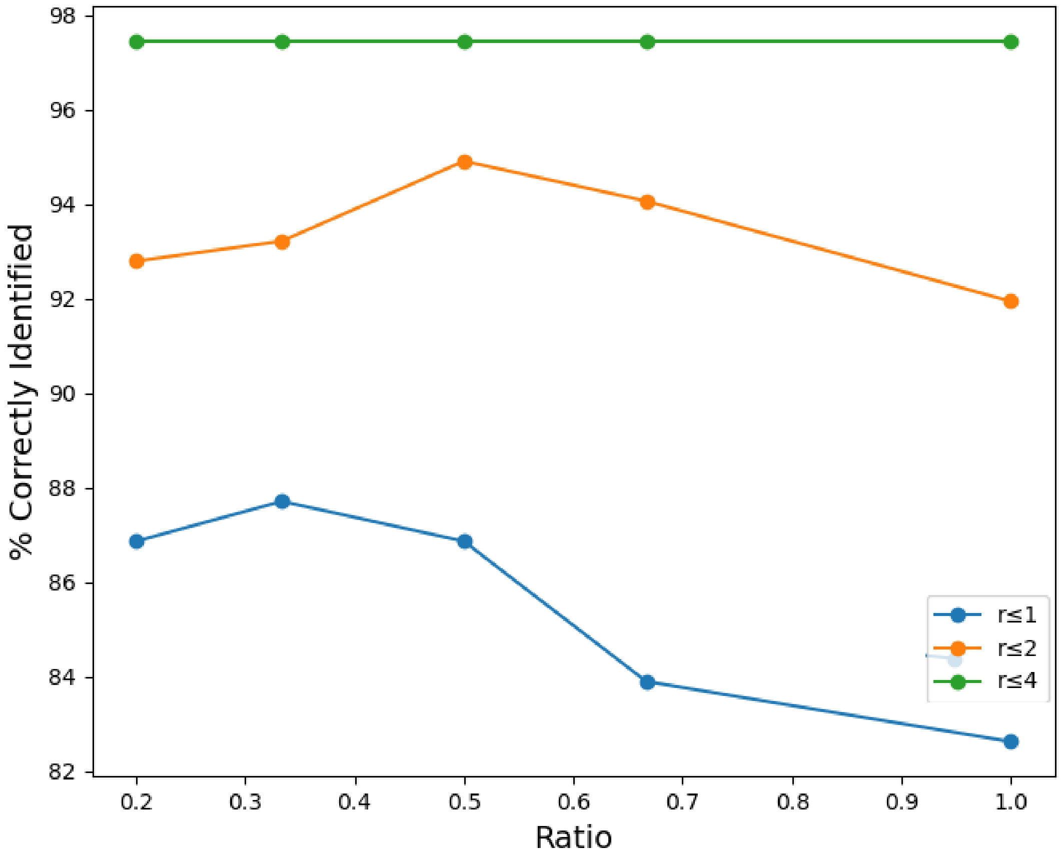

Figure 2: The rate of obtaining a rank r of 1, ≤ 2 and ≤ 4 for the s_{LBJ} scoring function as a function of δ_1 and the ratio $\delta_{2\ldots N_{\mathrm{peak}}}/\delta_1$. We observe only a weak dependence in the success rate, and there is no great advantage to using a different δ value to make likely candidate formula lists for fragment masses, as opposed to the parent peak.

2 Identifying Individual Compounds From "Mixed" CASMI Spectra

2.1 Testing Details

From the CASMI-2016 data set, we randomly sampled 100 pairs of mass spectra (with replacement) subject to the constraint that the molecular mass of either compound in the pair can be found in the mass spectrum and is not above some threshold $M_{\max}$, and that neither mass spectra is comprised of just a single mass peak. Each pair of mass spectra is first normalised based on the total intensity of all peaks in the mass spectrum, then combined together into a "mixed" mass spectrum. Two masses in the spectra are combined if they are within 10ppm of each other, and then corresponding intensities combined. The alphabet we chose for the testing is CHNO+, where the + denotes elements which are contained in either one of the two compounds. M_{upper} and M_{lower} corresponds to the maximum and minimum mass of the combined mass spectrum, respectively.

For testing purposes, we defined "correctly separated" as meeting the following two conditions:

1. The highest scoring parent candidate formula corresponds to the correct formula of one of the two compounds.
2. The next highest scoring parent candidate formula that is *not* a subformula of the first parent candidate formula corresponds to the correct formula of the other compound.

Condition 2 is necessary, large fragments of a given analyte also rank highly, given that the analyte formulae ranks highly. However, they can be easily identified and eliminated provided that the molecular formula of one of the compounds is not a subformula of the other.

We evaluated the performance of our method for $M_{\max}$ values 150, 200, 250, 300, 350 and 400.

2.2 Results

Although our method is able to in some cases successfully resolve the two distinct components of a mass spectra, the performance fell quite quickly as the maximum allowed mass increased (see Figure 3) The same trend occurs, but slightly more strongly, when the samples where one of the compounds is a subformula of the other is removed, given that two random compounds in CASMI are more likely to be subformulae if they are both smaller in size.

This result is expected, given that the number of parent candidate formulae increases quite rapidly as a function of the mass of the molecular ion. In order to successfully resolve the two components, our method needs to rank both compounds higher than any alternate parent candidate formula for either compound, or spurious parent candidate formulae that can explain portions of both spectra in the component. Another key limitation of our method is that it cannot be used to resolve the components if the molecular formula of one of the candidates is a subformula of the other; our method will simply treat that candidate as a fragment of the first candidate.

Nevertheless, our results show that if one places suitable restrictions on the allowed elements, are investigating compounds with very low molecular mass, or possess a very high resolution mass spectrometer, then it is possible to resolve co-eluting compounds using our method, provided they are not subformulae of each other. In fact, we show an example of this in practice for Compound 10 in the ORCHID data set. However, our method is not specifically designed or optimised to resolve co-eluting compounds in mass spectra. Further investigation is required in order to adapt PSG construction and scoring specifically for this purpose.

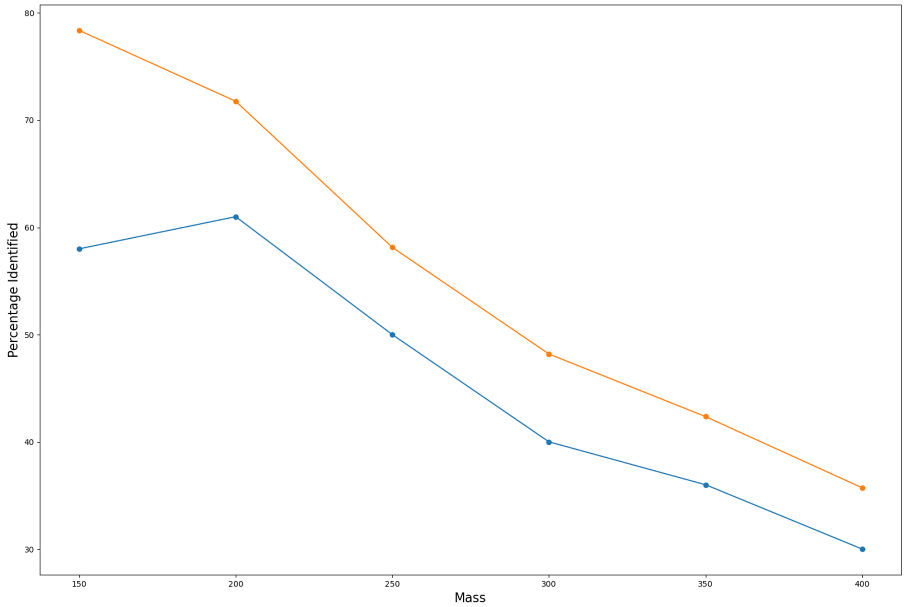

Figure 3: Percentage of components resolved in a randomly sampled (with replacement) of 100 pairs of mass spectra from the CASMI-2016 data set, combined into a mixed mass spectra, over all samples (blue) and all samples where neither compound is a subformula of the other.

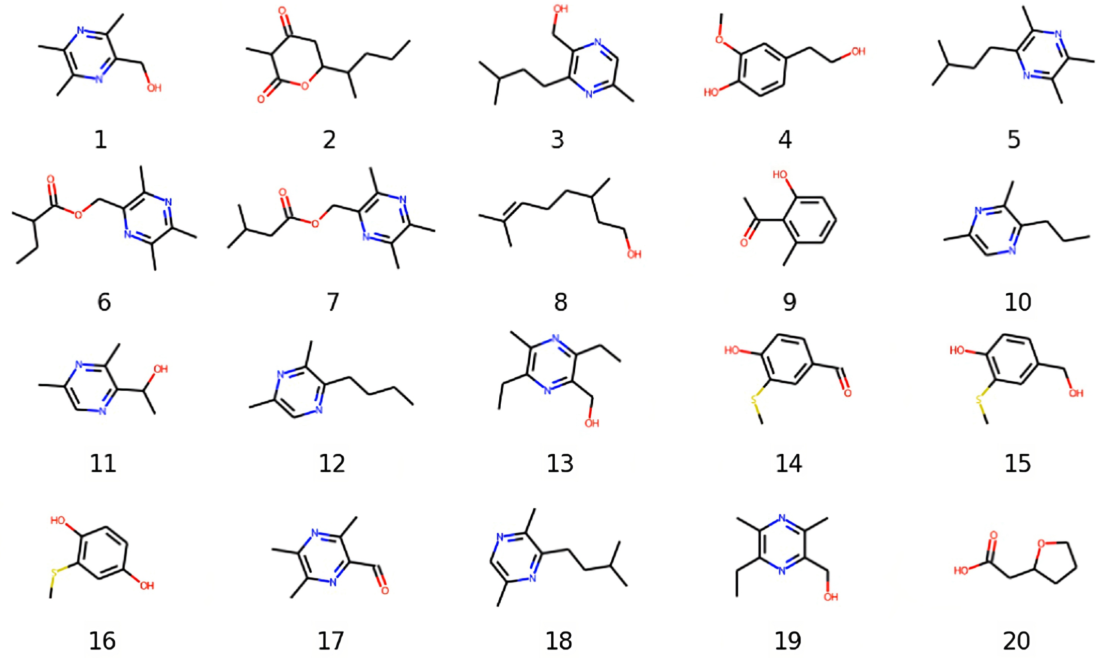

Figure 4: The twenty molecules comprising the orchid data set with their associated numberings (IDs). Names for these compounds are given in the supplementary material. Note that there are two spectra for compound 1, 14 and 15; referred to as 1(a) and 1(b), 14(a) and 14(b), and 15(a) and 15(b) respectively, for a total of 23 spectra in this data set.

3 ORCHID Data Set – Analysis Results

Table 1: The performance of both the product score and the edge score on the semiochemical dataset, as measured by the ranking of the correct molecular formula based on the scores. An asterisk is placed next to a rank when the molecular ion does not exist and a large fragment ion was annotated instead (Compound 18).

			Rank		
ID	Formula	Mass	s_{LBJ}	s_{ne}	s_v
1[a]	$C_8H_{12}N_2O$	152.095	3	5	6
1[b]	$C_8H_{12}N_2O$	152.095	1	1	1
2	$C_{11}H_{18}O_3$	198.126	1	1	2
3	$C_{11}H_{18}N_2O$	194.142	2	2	2
4	$C_9H_{12}O_3$	168.079	3	5	7
5	$C_{12}H_{20}N_2$	192.163	1	2	4
6	$C_{13}H_{20}N_2O_2$	236.152	1	2	5
7	$C_{13}H_{20}N_2O_2$	236.152	9	11	25
8	$C_{10}H_{20}O$	156.151	1	1	1
9	$C_9H_{10}O_2$	150.068	4	4	7
10	$C_9H_{14}N_2$	150.116	1	8	23
11	$C_8H_{12}N_2O$	152.095	1	1	12
12	$C_{10}H_{16}N_2$	164.131	4	21	31
13	$C_{10}H_{16}N_2O$	180.126	1	1	2
14[a]	$C_8H_8O_2S$	168.025	4	10	13
14[b]	$C_8H_8O_2S$	168.025	2	4	12
15[a]	$C_8H_{10}O_2S$	170.040	17	21	21
15[b]	$C_8H_{10}O_2S$	170.040	1	1	5
16	$C_7H_8O_2S$	156.025	3	6	14
17	$C_8H_{10}N_2O$	150.079	4	6	8
18	$C_{11}H_{18}N_2$	178.147	1*	2*	5*
19	$C_9H_{14}N_2O$	166.111	1	1	1
20	$C_6H_{10}O_3$	130.063	16	30	34

Rank	Formula	Monoisotopic Mass	Score	Spectrum Explained (%)
1	$C_{10}H_{13}N_5O$	219.112	0.201	76.34
2	$C_8H_{21}N_5O_2$	219.17	0.198	78.01
3	$C_8H_{12}N_2O$	152.095	0.192	72.19
4	$C_8H_{17}N_3O_2S$	219.104	0.186	77.81
5	$C_{13}H_{21}N_3$	219.174	0.18	52.27

Table 2: A list of candidate molecular formulae annotated to the mass spectrum of Compound 1(a). The correct molecular formula is $C_8H_{12}N_2O$, and its rank is 3 using the product score metric.

Rank	Formula	Monoisotopic Mass	Score	Spectrum Explained (%)
1	$C_8H_{12}N_2O$	152.095	0.18	68.84
2	$C_8H_{12}N_2$	136.1	0.152	36.16
3	$C_9H_{18}O_2$	158.131	0.15	22.28
4	$C_6H_{11}N_5$	153.101	0.132	33.16
5	$C_8H_{10}O_2$	138.068	0.121	18.44

Table 3: A list of candidate molecular formulae annotated to the mass spectrum of Compound 1(b). The correct molecular formula is $C_8H_{12}N_2O$, and its rank is 1 using the product score metric.

Rank	Formula	Monoisotopic Mass	Score	Spectrum Explained (%)
1	$C_{11}H_{18}O_3$	198.126	0.285	89.85
2	$C_{11}H_{18}O_2$	182.131	0.269	66.34
3	$C_{10}H_{18}O_2$	170.131	0.243	65.71
4	$C_{11}H_{16}O_2$	180.115	0.228	65.95
5	$C_{11}H_{25}NOS$	219.166	0.222	46.91

Table 4: A list of candidate molecular formulae annotated to the mass spectrum of Compound 2. The correct molecular formula is $C_{11}H_{18}O_3$, and its rank is 1 using the product score metric.

Rank	Formula	Monoisotopic Mass	Score	Spectrum Explained (%)
1	$C_{11}H_{20}N_2O$	196.158	0.19	45.41
2	$C_{11}H_{18}N_2O$	194.142	0.181	45.22
3	$C_{11}H_{16}N_2O$	192.126	0.174	44.41
4	$C_{10}H_{16}N_2O$	180.126	0.164	44.13
5	$C_{11}H_{18}N_2$	178.147	0.156	31.55

Table 5: A list of candidate molecular formulae annotated to the mass spectrum of Compound 3. The correct molecular formula is $C_{11}H_{18}N_2O$, and its rank is 2 using the product score metric.

Rank	Formula	Monoisotopic Mass	Score	Spectrum Explained (%)
1	$C_9H_{20}O_3$	176.141	0.157	95.14
2	$C_8H_8O_2$	136.052	0.143	20.36
3	$C_9H_{12}O_3$	168.079	0.137	94.02
4	$C_9H_{10}O$	134.073	0.127	13.79
5	$C_{11}H_{18}N_2O$	194.142	0.123	16.05

Table 6: A list of candidate molecular formulae annotated to the mass spectrum of Compound 4. The correct molecular formula is $C_9H_{12}O_3$, and its rank is 3 using the product score metric.

Rank	Formula	Monoisotopic Mass	Score	Spectrum Explained (%)
1	$C_{12}H_{20}N_2$	192.163	0.266	89.69
2	$C_{14}H_{20}N_2O$	232.158	0.26	95.59
3	$C_{11}H_{18}N_2$	178.147	0.25	89.44
4	$C_{10}H_{16}N_2$	164.131	0.234	86.02
5	$C_{10}H_{14}N_2$	162.116	0.218	85.57

Table 7: A list of candidate molecular formulae annotated to the mass spectrum of Compound 5. The correct molecular formula is $C_{12}H_{20}N_2$, and its rank is 1 using the product score metric.

Rank	Formula	Monoisotopic Mass	Score	Spectrum Explained (%)
1	$C_{13}H_{20}N_2O_2$	236.152	0.259	93.32
2	$C_{10}H_{24}N_2O_2S$	236.156	0.245	94.55
3	$C_{12}H_{20}N_4O$	236.164	0.237	88.95
4	$C_9H_{15}N_7$	221.139	0.22	90.27
5	$C_8H_{19}N_3O_4$	221.138	0.219	92.7

Table 8: A list of candidate molecular formulae annotated to the mass spectrum of Compound 6. The correct molecular formula is $C_{13}H_{20}N_2O_2$, and its rank is 1 using the product score metric.

Rank	Formula	Monoisotopic Mass	Score	Spectrum Explained (%)
1	$C_9H_{14}N_2$	150.116	0.085	13.04
2	$C_{13}H_{22}N_2O_2$	238.168	0.084	17.43
3	$C_9H_{12}N_2$	148.1	0.081	12.99
4	$C_{11}H_{19}N_5O$	237.159	0.08	50.05
5	$C_9H_{18}N_8$	238.165	0.08	50.0
6	$C_8H_{12}N_2$	136.1	0.077	12.99
7	$C_{11}H_{17}N_5O$	235.143	0.076	48.71
8	$C_{12}H_{18}N_2O_2$	222.137	0.075	16.07
9	$C_{13}H_{20}N_2O_2$	236.152	0.075	17.28

Table 9: A list of candidate molecular formulae annotated to the mass spectrum of Compound 7. The correct molecular formula is $C_{13}H_{20}N_2O_2$, and its rank is 9 using the product score metric.

Rank	Formula	Monoisotopic Mass	Score	Spectrum Explained (%)
1	$C_{10}H_{20}O$	156.151	0.301	73.79
2	$C_{10}H_{18}$	138.141	0.287	66.85
3	$C_9H_{19}NO$	157.147	0.285	69.98
4	$C_9H_{17}N$	139.136	0.273	64.46
5	$C_{10}H_{16}$	136.125	0.264	63.97

Table 10: A list of candidate molecular formulae annotated to the mass spectrum of Compound 8. The correct molecular formula is $C_{10}H_{20}O$, and its rank is 1 using the product score metric.

Rank	Formula	Monoisotopic Mass	Score	Spectrum Explained (%)
1	$C_9H_{18}O$	142.136	0.256	37.63
2	$C_9H_{12}O_2$	152.084	0.246	86.98
3	$C_9H_{10}O_3$	166.063	0.208	82.73
4	$C_9H_{10}O_2$	150.068	0.2	82.62
5	$C_7H_{17}NS_2$	179.08	0.185	29.23

Table 11: A list of candidate molecular formulae annotated to the mass spectrum of Compound 9. The correct molecular formula is $C_9H_{10}O_2$, and its rank is 4 using the product score metric.

Rank	Formula	Monoisotopic Mass	Score	Spectrum Explained (%)
1	$C_9H_{14}N_2$	150.116	0.179	42.88
2	$C_8H_{12}N_2$	136.1	0.155	41.15
3	$C_{14}HNO_3$	230.996	0.151	20.75
4	$C_9H_{12}N_4O$	192.101	0.143	49.79
5	$C_9HN_3O_5$	230.992	0.14	24.62

Table 12: A list of candidate molecular formulae annotated to the mass spectrum of Compound 10. The correct molecular formula is $C_9H_{14}N_2$, and its rank is 1 using the product score metric.

Rank	Formula	Monoisotopic Mass	Score	Spectrum Explained (%)
1	$C_8H_{12}N_2O$	152.095	0.267	93.69
2	$C_9H_{13}N_7$	219.123	0.26	93.75
3	$C_{10}H_{16}N_2$	164.131	0.25	49.7
4	$C_{11}H_{11}N_7$	241.108	0.247	93.41
5	$C_8H_{12}N_2$	136.1	0.239	49.7

Table 13: A list of candidate molecular formulae annotated to the mass spectrum of Compound 11. The correct molecular formula is $C_8H_{12}N_2O$, and its rank is 1 using the product score metric.

Rank	Formula	Monoisotopic Mass	Score	Spectrum Explained (%)
1	$C_6H_{13}NO_3$	147.09	0.109	14.91
2	$C_6H_{15}NO_3$	149.105	0.109	15.21
3	$C_9H_{14}N_2$	150.116	0.095	4.9
4	$C_{10}H_{16}N_2$	164.131	0.095	4.91
5	$C_6H_9NO_5S$	207.02	0.095	13.79

Table 14: A list of candidate molecular formulae annotated to the mass spectrum of Compound 12. The correct molecular formula is $C_{10}H_{16}N_2$, and its rank is 4 using the product score metric.

Rank	Formula	Monoisotopic Mass	Score	Spectrum Explained (%)
1	$C_{10}H_{16}N_2O$	180.126	0.284	62.12
2	$C_{10}H_{16}N_2$	164.131	0.262	39.09
3	$C_{10}H_{14}N_2$	162.116	0.233	33.43
4	$C_9H_{14}N_2O$	166.111	0.228	33.72
5	$C_8H_{15}N_5$	181.133	0.222	39.63

Table 15: A list of candidate molecular formulae annotated to the mass spectrum of Compound 13. The correct molecular formula is $C_{10}H_{16}N_2O$, and its rank is 1 using the product score metric.

Rank	Formula	Monoisotopic Mass	Score	Spectrum Explained (%)
1	$C_{11}H_{25}NOS$	219.166	0.172	40.9
2	$C_7H_8O_4S_2$	219.986	0.169	45.29
3	$C_7H_{18}N_2O_2$	162.137	0.166	22.25
4	$C_8H_8O_2S$	168.025	0.165	76.4
5	$C_{12}H_{23}NS$	213.155	0.162	30.05

Table 16: A list of candidate molecular formulae annotated to the mass spectrum of Compound 14(a). The correct molecular formula is $C_8H_8O_2S$, and its rank is 4 using the product score metric.

Rank	Formula	Monoisotopic Mass	Score	Spectrum Explained (%)
1	$C_{10}H_{17}NO_3S$	231.093	0.23	74.99
2	$C_8H_8O_2S$	168.025	0.222	60.94
3	$C_7H_8O_2S$	156.025	0.193	45.11
4	$C_7H_7NO_2S$	169.02	0.189	40.23
5	$C_7H_{17}N_5OS$	219.115	0.173	70.77

Table 17: A list of candidate molecular formulae annotated to the mass spectrum of Compound 14(b). The correct molecular formula is $C_8H_8O_2S$, and its rank is 2 using the product score metric.

Rank	Formula	Monoisotopic Mass	Score	Spectrum Explained (%)
1	$C_{12}H_{24}O_2$	200.178	0.238	71.88
2	$C_{10}H_{23}N_3O$	201.184	0.214	75.44
3	$C_9H_{20}N_2O_3S$	236.119	0.21	80.06
4	$C_{10}H_{20}O_6$	236.126	0.207	66.47
5	$C_8H_{19}NO_2S$	193.114	0.204	71.12
6	$C_9H_{18}O_2$	158.131	0.203	60.73
7	$C_{14}H_{20}OS$	236.123	0.184	41.32
8	$C_{11}H_{24}OS_2$	236.127	0.184	41.16
9	$C_8H_{17}N_3O$	171.137	0.174	63.61
10	$C_8H_{20}N_6$	200.175	0.169	55.81
11	$C_{10}H_{15}N_3O$	193.122	0.168	70.92
12	$C_9H_{20}N_2O$	172.158	0.168	37.04
13	$C_7H_{19}N_3OS$	193.125	0.167	74.28
14	$C_{11}H_{16}N_4O_2$	236.127	0.167	77.64
15	$C_8H_{20}N_4O_2S$	236.131	0.167	82.36
16	$C_{17}H_{16}O$	236.12	0.165	35.96
17	$C_8H_{10}O_2S$	170.04	0.148	52.33

Table 18: A list of candidate molecular formulae annotated to the mass spectrum of Compound 15(a). The correct molecular formula is $C_8H_{10}O_2S$, and its rank is 17 using the product score metric.

Rank	Formula	Monoisotopic Mass	Score	Spectrum Explained (%)
1	$C_{10}H_{17}NO_3S$	231.093	0.23	74.99
2	$C_8H_8O_2S$	168.025	0.222	60.94
3	$C_7H_8O_2S$	156.025	0.193	45.11
4	$C_7H_7NO_2S$	169.02	0.189	40.23
5	$C_7H_{17}N_5OS$	219.115	0.173	70.77

Table 19: A list of candidate molecular formulae annotated to the mass spectrum of Compound 16. The correct molecular formula is $C_7H_8O_2S$, and its rank is 3 using the product score metric.

Rank	Formula	Monoisotopic Mass	Score	Spectrum Explained (%)
1	$C_8H_{10}O_2S$	170.04	0.216	84.01
2	$C_8H_{10}OS$	154.045	0.193	53.82
3	$C_{12}H_{24}O_2$	200.178	0.191	76.07
4	$C_{13}H_{17}NO_2$	219.126	0.186	77.7
5	$C_{10}H_{19}NO_2$	185.142	0.179	49.41

Table 20: A list of candidate molecular formulae annotated to the mass spectrum of Compound 15(b). The correct molecular formula is $C_8H_{10}O_2S$, and its rank is 1 using the product score metric.

Rank	Formula	Monoisotopic Mass	Score	Spectrum Explained (%)
1	$C_7H_{13}N_3O_5$	219.086	0.224	86.01
2	$C_8H_{17}N_3S_2$	219.086	0.214	61.88
3	$C_{14}H_9N_3$	219.08	0.21	43.65
4	$C_8H_{10}N_2O$	150.079	0.209	77.9
5	$C_8H_9N_7O$	219.087	0.208	69.77

Table 21: A list of candidate molecular formulae annotated to the mass spectrum of Compound 17. The correct molecular formula is $C_8H_{10}N_2O$, and its rank is 4 using the product score metric.

Rank	Formula	Monoisotopic Mass	Score	Spectrum Explained (%)
1	$C_{10}H_{16}N_2$	164.131	0.247	78.17
2	$C_9H_{12}N_2$	148.1	0.228	73.0
3	$C_9H_{14}N_2$	150.116	0.228	73.0
4	$C_9H_{13}N_7$	219.123	0.224	73.19
5	$C_8H_{12}N_2$	136.1	0.197	72.39

Table 22: A list of candidate molecular formulae annotated to the mass spectrum of Compound 18. The correct molecular formula is $C_{11}H_{18}N_2$. Although the molecular ion was not detected in the mass spectrum analysed, the rank of a large fragment ion, $C_{10}H_{16}N_2$ is 1 using the product score metric.

Rank	Formula	Monoisotopic Mass	Score	Spectrum Explained (%)
1	$C_9H_{14}N_2O$	166.111	0.201	59.42
2	$C_9H_{14}N_2$	150.116	0.18	38.4
3	$C_8H_{13}N_3O$	167.106	0.153	36.47
4	$C_8H_{12}N_2O$	152.095	0.146	29.16
5	$C_9H_{12}N_2$	148.1	0.139	28.8

Table 23: A list of candidate molecular formulae annotated to the mass spectrum of Compound 19. The correct molecular formula is $C_9H_{14}N_2O$, and its rank is 1 using the product score metric.

Rank	Formula	Monoisotopic Mass	Score	Spectrum Explained (%)
1	$C_7H_{15}N_3O_3S$	221.083	0.146	58.27
2	$C_8H_{17}NO_3S_2$	239.065	0.144	48.48
3	$C_{10}H_{13}N_3O_2S$	239.073	0.143	59.95
4	$C_6H_8N_2O_3S_2$	219.998	0.135	55.04
5	$C_{10}H_{11}N_3O_3$	221.08	0.133	50.08
6	$C_{11}H_{13}NO_3S$	239.062	0.133	50.16
7	$C_5H_{13}N_5O_4S$	239.069	0.132	60.78
8	$C_{13}H_9N_3O_2$	239.069	0.132	50.75
9	$C_8H_{15}NO_6$	221.09	0.128	39.45
10	$C_9H_{11}N_5O_2$	221.091	0.125	50.08
11	$C_7H_{13}NO_8$	239.064	0.123	38.95
12	$C_8H_9N_5O_4$	239.065	0.122	58.78
13	$C_9H_{19}NOS_2$	221.091	0.119	42.29
14	$C_{12}H_{15}NOS$	221.087	0.11	43.97
15	$C_5H_8N_4S_3$	219.991	0.107	18.84
16	$C_6H_{10}O_3$	130.063	0.103	30.08

Table 24: A list of candidate molecular formulae annotated to the mass spectrum of Compound 20. The correct molecular formula is $C_6H_{10}O_3$, and its rank is 16 using the product score metric.

4 ORCHID Data Set – Example Usage of 2DFPs

This section details three examples of how representing the Parent Subformula Graph (PSG) as the 2-Dimensional Fragment Plot (2DFP) may aid the experimentalist in determining the correct molecular formula of the analyte.

4.1 Low S/N - Mass spectra of minor GC peaks: Compound 10

Our method can extract information from a mass spectrum, which could be used to identify compounds even when the corresponding total ion chromatogram peak for said compound is extremely weak and difficult to distinguish from background noise. To illustrate this, we examine the mass spectra of Compound 10, possessing the molecular formula $C_9H_{14}N_2$. Due to the presence of only trace amounts of the compound and/or low ionisation efficiency, there is no visible presence of a chromatographic peak (Figure 5 B). In this situation, the analyst will typically need to manually trace scan by scan through the chromatogram in order to monitor the increase or decrease of certain ion masses hypothesised to be the molecular ion, or a key fragment ion. This is very time consuming, requires considerable specialist knowledge regarding which masses are more or less likely to be notable, and subject to confirmation bias. The molecular ion in this case (m/z = 150) is barely visible, while several other background ions are more abundant (i.e. m/z = 164, 169 and 200) (Figure 5 B). However, our method allows the immediate identification of the molecular formula $C_9H_{14}N_2$ as the top ranked candidate (Figure 6).

4.2 Overlapping chromatographic peaks: Compound 12

We also present as an example the identification of Compound 12, possessing the chemical formula $C_{10}H_{16}N_2$. In this case, the compound is co-eluting with another compound, proposed to be hydroquinone ($C_6H_6O_2$, M = 110), whose molecular ion and fragments are considerably interfering with the mass spectrum of the target compound (Figure 5 A, C). Nonetheless, the annotation containing the correct molecular formula still obtained $r = 3$ using s_{LBJ}, compared to a $r = 30$ using s_{v}. Of the highest scoring parent formula ($C_6H_{15}NO_3$ see Table 13 in the Supplementary Information), examination of its 2DFPs reveals that although the formula possess a very low RDBE, there exists a number of fragment formulae in the annotation which possess spuriously high RDBEs, corresponding to theoretical

losses of neutral fragments containing many hydrogen atoms (in the above example, H_9O for the first formula). The second highest scoring parent formula ($C_6H_{13}NO_3$) possesses a similar feature. In contrast, the 2DFP of the correct parent formula does not exhibit this feature (see Figure 7).

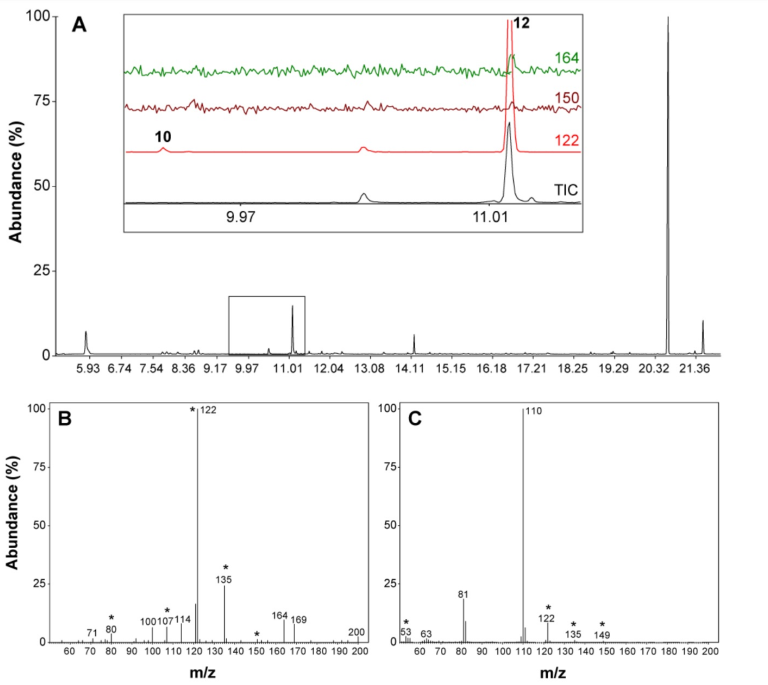

Figure 5: A. Total ion chromatogram (TIC) and extracted ions for the molecular ions (m/z = 150, 164) and base peak (m/z = 122) for compounds 10 and 12 in a floral extract of Drakaea glyptodon. The molecular ion for 10 (m/z = 150) is not visible in the extracted ion chromatogram (inset). B. Mass spectrum for the chromatographic peak of compound 10 (five scans across the peak, with background subtraction). Ions corresponding to the target compound are marked with an asterisk. C. Mass spectrum for the chromatographic peak of compound 12 (five scans across the peak, with background subtraction). Ions corresponding to the target compound are marked with an asterisk. The two main ions from the co-eluting hydroquinone (m/z = 81, 110) are the dominant ions across the selected scans.

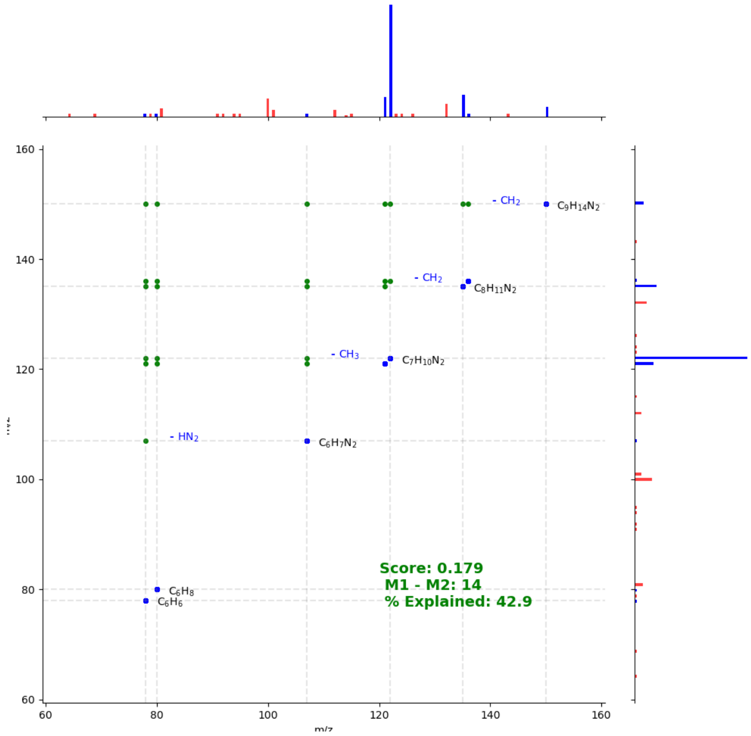

Figure 6: The 2DFP plot with mass peak $M_1 = 150$ annotated by formula $C_9H_{14}N_2$. Despite the presence of numerous noise peaks, due to the very small peak height in the mass chromatogram, the formula alongside a number of fragments with sensible (possible) neutral losses can be seen, suggesting this to be a likely molecular formula for the compound.

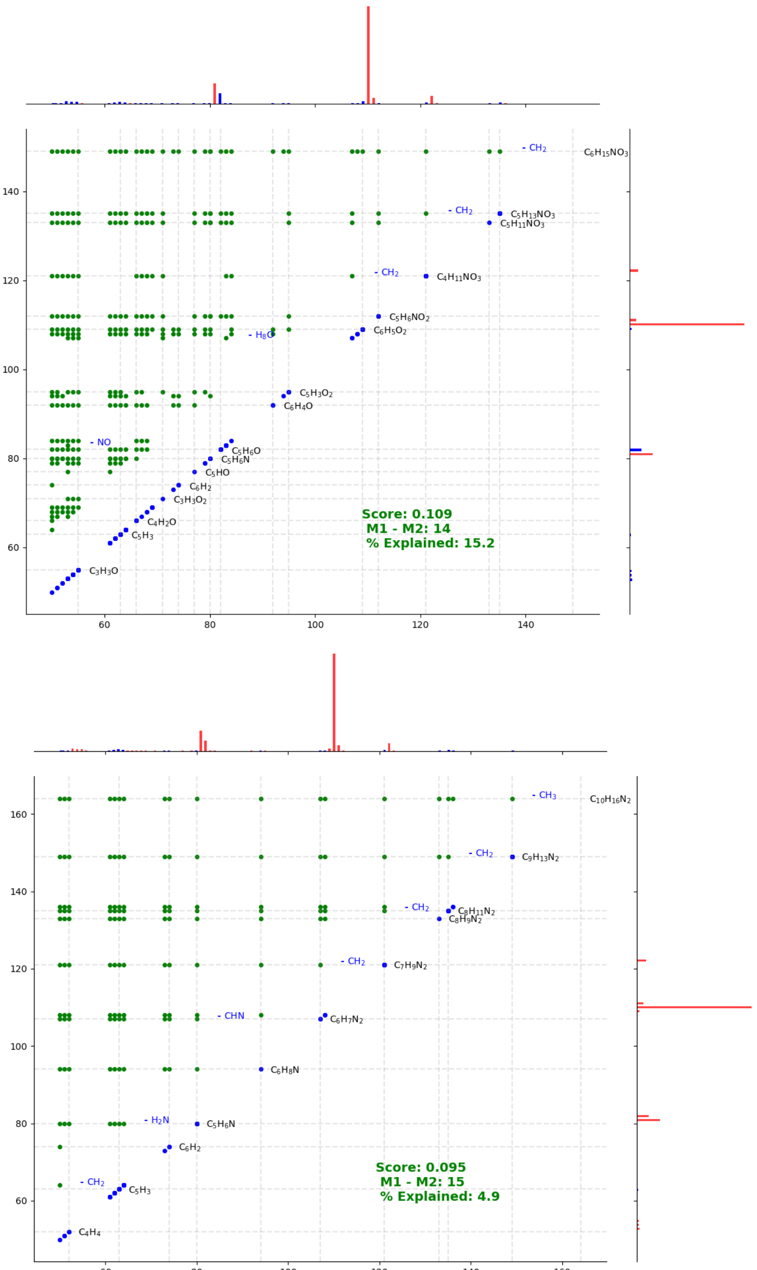

Figure 7: 2DFP generated from the PSG derived from the highest scoring (incorrect) parent candidate formula, $C_6H_{15}NO_3$ (top) and from the correct parent candidate formula, $C_{10}H_{16}N_2$ (bottom). The very high intensity mass peak at $M = 110$ corresponds to the molecular ion of the co-eluting hydroquinone.
